# Women’s experiences of psychological treatment and psychosocial interventions for postpartum depression: a qualitative systematic review and meta-synthesis

**DOI:** 10.1186/s12905-023-02772-8

**Published:** 2023-11-14

**Authors:** Pamela Massoudi, Leif A. Strömwall, Johan Åhlen, Maja Kärrman Fredriksson, Anna Dencker, Ewa Andersson

**Affiliations:** 1 Department of Psychology, University of Gothenburg, Gothenburg, Sweden; 2 Department of Research and Development, Region Kronoberg, Växjö, Sweden; 3 Swedish Agency for Health Technology Assessment and Assessment of Social Services, Stockholm, Sweden; 4 Department of Global Public Health, Karolinska Institutet, Stockholm, Sweden; 5 Institute of Health and Care Sciences, The Sahlgrenska Academy, University of Gothenburg, Gothenburg, Sweden; 6 Department of Women’s and Children’s Health, Karolinska Institutet, Stockholm, Sweden

**Keywords:** Perinatal mental health, Postnatal depression, Support, Psychotherapy, Treatment, Mothers, GRADE-CERQual, Meta-synthesis

## Abstract

**Background:**

To provide a comprehensive, systematic evaluation of the literature on experiences of psychological interventions for postpartum depression (PPD) in women. Depression is one of the most common postpartum mental disorders. Studies have identified that psychological interventions reduce depressive symptoms. However, less is known about the experiences of women who have received such treatments.

**Methods:**

A systematic review of the literature was conducted by searching five databases (CINAHL, Cochrane Library, EMBASE, Medline, PsycINFO), in August 2022. Studies with qualitative methodology examining women’s experiences of professional treatment for PPD were included and checked for methodological quality. Eight studies (total N = 255) contributed to the findings, which were synthesized using thematic synthesis. Confidence in the synthesized evidence was assessed with GRADE CERQual.

**Findings:**

The women had received cognitive behavioral therapy (5 studies) or supportive home visits (3 studies). Treatments were individual or group-based. Two main themes were identified: Circumstances and expectations, and Experiences of treatment, with six descriptive themes. Establishing a good relationship to their health professional was important for the women, regardless of treatment model. They also expressed that they wanted to be able to choose the type and format of treatment. The women were satisfied with the support and treatment received and expressed that their emotional well-being had been improved as well as the relationship to their infant.

**Conclusion:**

The findings can be helpful to develop and tailor patient-centered care for women who are experiencing postnatal depression.

**Supplementary Information:**

The online version contains supplementary material available at 10.1186/s12905-023-02772-8.

## Background

Pregnancy and the first year after childbirth involve significant changes in a woman’s life and can be associated with emotional distress of varying types and degrees. For some, worry and mood disturbances are natural and transient reactions to the challenges of a new life situation. For others, symptoms can persist and develop into a condition where support or treatment is needed. Depression is one of the most common postpartum mental disorders during this period. The prevalence of postpartum depression (PPD) has been estimated at 5–9% in high income countries, around 13% when self-report measures are used [1, 2]. Women with previous mental health problems are more at risk, as well as women with previous or current stressful life experiences, especially being exposed to interpersonal violence, partner relationship problems, migration, and lack of support [3, 4]. Associations between PPD and adverse outcomes on the child are most evident when depression is severe or recurrent, or when associated risk factors may explain a substantial part of the negative outcome on children [3].

In general, and across various cultures, mothers with PPD have been found to prefer talking therapies or supportive interventions over pharmacological treatments, in part due to fear of negative effects on the child by transmission to breastmilk [5–7]. A recent review highlighted how mothers put what they thought was best for their baby first when making decisions about treatment, including taking or not taking medication [8].

Systematic reviews have found that psychotherapy and psychosocial interventions for perinatal depression are generally effective [9–11]. Common treatments for PPD are cognitive behavior therapy (CBT), interpersonal psychotherapy (IPT), and non-directive supportive counseling, also called listening visits [9]. Treatments can use an individual or group format, take place as home visits, at a clinic, or be internet-based, and are often tailored for the postnatal period, sometimes including a parent-child interaction component.

Besides outcomes in terms of symptom reduction, it is also relevant to explore women’s experiences of treatment. A meta-synthesis focusing on experiences of seeking and receiving psychosocial interventions for postpartum depression found that women could experience several barriers to help-seeking, but that they were generally positive to the interventions they had received [12]. However, this meta-synthesis included low-quality studies. Barriers can be lack of time, stigma, childcare or transportation issues [5–7], and negative healthcare experiences [13]. Some women also have concerns about being judged as a “bad mother”, which may delay seeking help. Another meta-synthesis of studies concerning the experiences of perinatal women with a broader range of mental health problems, identified several unmet needs of information, collaborative integrated care, and post-treatment follow-up [14]. Some important components of treatment expressed by the women were the importance of the health professionals’ non-judgmental attitude as well as conveying hope.

The aim of the current review was to provide an updated and comprehensive understanding of women’s experiences of psychological interventions for postpartum depression, based on a systematic evaluation of the literature and a meta-synthesis of the findings, including an assessment of the reliability of the findings.

## Methods

### Search strategy

An information specialist (MKF) searched five databases: CINAHL (EBSCO), Cochrane Library (Wiley), EMBASE (Embase.com), Medline (Ovid), PsycINFO (EBSCO). Searches were run in November and December 2021, and updates in June 2022. A manual search of reference lists from the included articles was also undertaken to identify studies not captured by the electronic search.

The search strategy was developed by the information specialist in collaboration with the experts in the review team, and combined terms and phrases describing the population, interventions, patients’ experiences, and qualitative research methods. Another information specialist at the Swedish Agency for Health Technology Assessment and Assessment of Social Services (SBU) reviewed the search strategy using the PRESS Checklist [15]. The search strategy and search terms used can be found in Appendix 1. The review used PRISMA Guidelines for reporting the search strategy [16].

### Inclusion criteria

Studies were included if they satisfied the inclusion criteria, see Table 1.

**Table 1:** Inclusion and exclusion criteria

	Inclusion	Exclusion
Setting	Outpatient care Studies conducted in high-income countries	
Population	Women who had received psychological treatment for postnatal depression initiated within 12 months after the birth of the child	Studies including women with psychotic disorders, bipolar disorder, or substance abuse
Intervention	Psychological treatments and supportive counselling	Supportive interventions that were not led by a professional
Evaluation	Experience of treatment for postnatal depression	Treatments with mixed prenatal and postnatal populations
Study design	Studies with qualitative or mixed methods methodology examining experiences of treatment for PPD	
Publication languages	English, Danish, Norwegian, or Swedish	
Publication year	Studies published in 1995 and thereafter.	

### Study selection

**Fig. 1:**
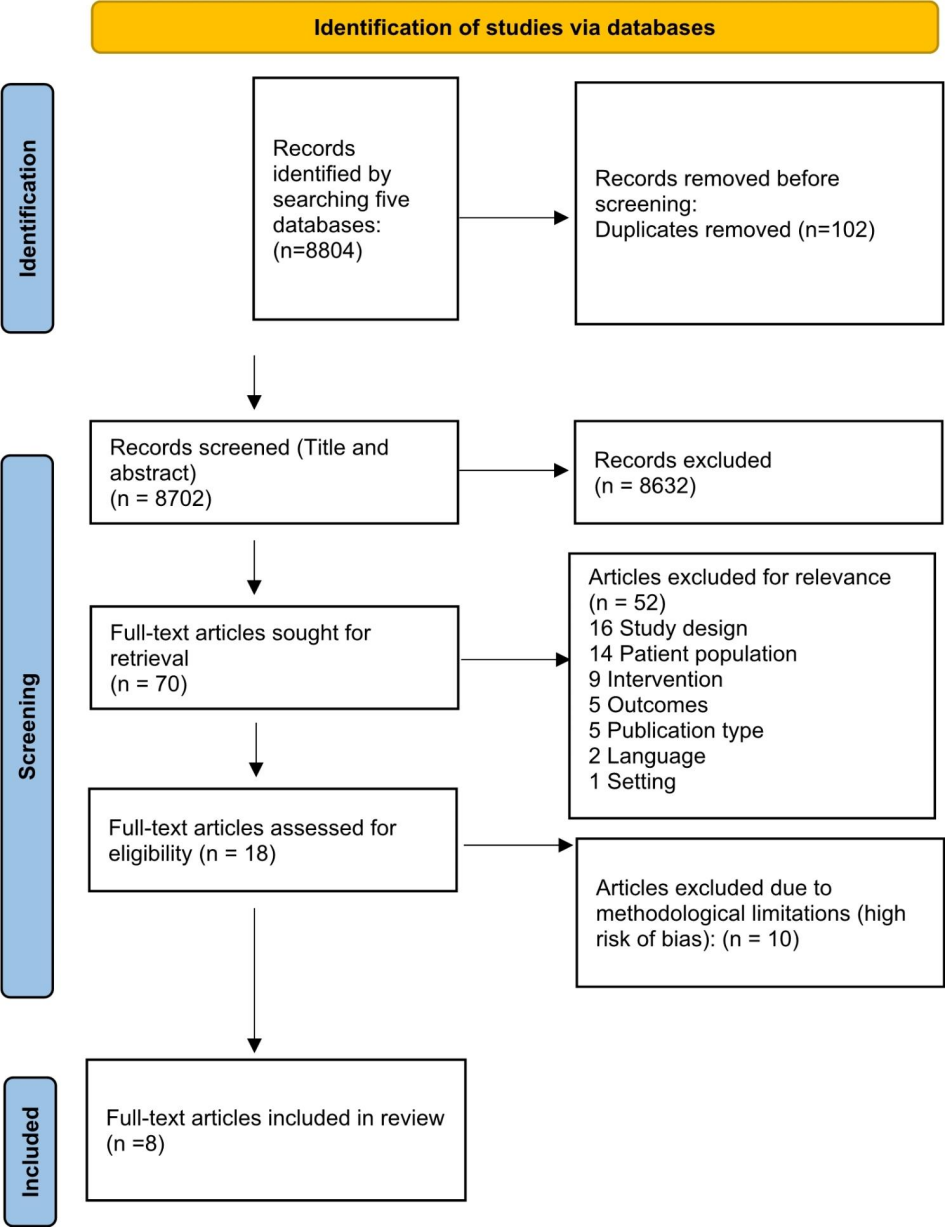
PRISMA Flow of study selection process

The search process yielded 8804 unique studies. All titles and abstracts were screened for eligibility, 70 articles were assessed in full-text, and eight studies were included for data extraction and synthesis after assessing for quality (Fig. 1).

### Quality assessment of primary studies

To assess the methodological quality and risk of bias, included studies were evaluated using the SBU Quality assessment tool for studies with a qualitative design [17]. This critical appraisal tool consists of five domains (adherence to epistemological position, recruitment and appropriateness of participants, appropriateness of data collection procedures, aspects of the data analysis, and the role of the researcher), each with signaling questions.

Three authors initially assessed each study (LS, PM, AD, EA, or JÅ), followed by a consensus discussion concerning the degree to which the methodological limitations impacted the findings, assessed as low, moderate, or high risk. For studies with a low or moderate risk of bias, data was extracted and compiled in tables while studies with high risk of bias were excluded from the further analyses.

### Data extraction and synthesis

An inductive thematic synthesis was conducted using a three-stage procedure, largely in line with Thomas and Harden (2008) [18].

First, the included studies were read, in depth, to provide a full understanding. Three authors (EA, PM, and LS) also discussed their respective pre-understanding of the field, with both insider and outsider perspectives. PM (clinical psychologist) and EA (midwife) are both researchers in the field; PM also had experience of treating PPD. LS is a psychology professor, not experienced in this field, but in research methodology. These authors then independently extracted meaningful units from the included studies and translated them into codes. In stage 2, codes were grouped into descriptive themes, first individually, and then in a consensus procedure until everyone agreed.

The same three authors grouped the stage 2 themes, resulting in two overarching stage 3 themes. Thomas and Harden (2008) [18] have described this third step as generating analytical themes. In the current synthesis, however, the two main themes generated were descriptive, and will therefore be referred to as main themes. Throughout the process, the emerging results were reflected upon in relation to the results of the primary studies to ensure that the findings would be grounded in the data and interrelated with each other to form a systematic whole. Quotes illustrating the findings were selected by all authors together.

### Assessment of the reliability of the combined findings

The reliability of the synthesis was assessed using GRADE-CERQual (www.cerqual.org), which consists of four domains: methodological limitations, coherence, adequacy of data, and relevance. Three authors (EA, PM, LS) conducted the assessments. First, two authors (LS and EA) assessed the synthesis individually and proposed a preliminary assessment which was then reviewed by a third author (PM), adding new perspectives. Finally, consensus was reached among the three authors to reach a reliability assessment for each descriptive theme in stage 2.

## Results

### Characteristics of the included studies

The eight studies represented the experiences of 255 women from the UK, Australia, and Canada. See Table 2 for detailed information about the participants, the treatments, and the research methodologies.

**Table 2:** Characteristics and methodological assessment of the included studies

First author, year, country	Aim of research	Participants	Setting, Intervention	Data collection	Analysis, Validation of the analysis	Methodological limitations
Hadfield 2019 [19] UK	Experiences of psychological interventions for PND^1^	14 women (mean age 32 years) who had received psychological treatment for PND.	NHS primary care mental health settings. CBT in 12 individual (6 cases) or 6 group sessions (8 cases). EMDR^2^ (1 case).	Semi-structured individual interviews.	Framework analysis. Five stages of analysis (Ritchie & Spencer, 1994).	Moderate. Some concerns for sampling strategy and lack of discussion on validation procedure.
Masood 2015 [20] UK	Acceptability and overall experience of the Positive Health Programme by British South Asian mothers.	17 women (20–45 years), all South Asian living in UK. .	Primary care: General Practices and Children’s Centres. Part of an RCT. Positive Health Programme: manual-based and culturally adapted group CBT, 12 sessions over 3 months.	In-depth individual interviews at home Iterative data collection until data saturation was achieved.	Thematic analysis. First at semantic level, then at latent level. Researcher and supervisor reviewed themes and subthemes.	Moderate. Some concerns for validation of analysis, and for lack of information about researcher’s position.
O’Mahen 2015 [21] UK	Patient perspectives on engagement and barriers to the Netmums “Helping with Depression” treatment.	22 women (mean age 31.3 years) within a year of receiving treatment.	Part of a large effectiveness trial. Online self-help behavioural activation. Minimum 11 sessions.	Individual semi-structured interviews (via phone). Interview guide was modified as data collection went on.	Thematic analysis, using principles of grounded theory. At least two independent coders. Continuous discussion and interpretation of data to reach group consensus. Coder agreement calculated (81%, acceptable).	Moderate. Some concerns regarding lack of information on researcher positionality.
Pugh 2014 [22] Canada	Experience of therapist-assisted internet CBT (TAICBT) for PPD.	24 women in the treatment arm of a clinical trial.	Part of a larger RCT program. TAICBT, 7 modules over 10 weeks.	Responses to 10 open-ended questions on an online survey.	Thematic analysis (Braun & Clarke, 2006). Two coders worked independently; a third experienced researcher was consulted on the analytic framework.	Moderate. Some concerns for lack of declaration of researcher positionality.
Rossiter 2012 [23] Australia	Experiences of a home visiting program (HVP).	111 women diagnosed as depressed. Questionnaire sent to all women who had received the HVP during four waves.	Primary care. Part of a large evaluation project on Home Visiting Programme. HVP including a video of mother-infant interaction, providing support and strengthening parental capacities. 10 visits.	Satisfaction questionnaire (both rating scales and open-ended). Hand-written answers to 14 open-ended questions were used as data.	Thematic content analysis. No specified validation method.	Moderate. Some concerns for the low response rate (~ 50%), lack of external validation, and lack of discussion on researcher positionality.
Shakespeare 2006 [24] UK	Experiences of listening visits for PND.	16 women (19–41 years).	Primary care, Oxford City. Part of a wider project evaluating women’s views on screening and listening visits. Listening Visits, usually 4 visits at weekly intervals.	Individual interviews following a topic guide, which was updated during the data collections phase to explore emerging themes.	Qualitative thematic analysis (Pope et al. 1999). All three authors coded text and discussed emerging themes. Interview quality was checked.	Moderate. Some concerns for low response rate and time between the visits and the interviews.
Slade 2010 [25] UK	Experiences of the identification and management of depression and acceptance of postnatal care by health visitors in the PoNDER trial.	30 women, 9 (control home visits), 10 (CBT) and 11 (PCA). Ages 18–45. Women in the trial with EPDS ≥ 18	Primary care. Part of a large RCT (PoNDER). Three interventions: Cognitive-behavioural approach, person-centred approach, treatment as usual (home visits). Up to 8 one-hour sessions.	Individual interviews, at participants’ home, following a semi-structured interview schedule.	A “template approach” (King, 1998) was used. Prespecified template of themes important to the research question, to answer specific questions. Three interviewers and coders developed codes together. Final themes and subthemes verified by two other researchers.	Moderate. Some concerns for lack of discussion on researcher position.
Turner 2009 [26] UK	Experiences of health visitor delivered listening visits (LV) as a treatment for PND.	22 women (19–45 years), clinically diagnosed with depression.	Primary care in three cities. Part of the RESPOND trial, an RCT evaluation. Listening Visits. 4–8 weekly visits.	Individual interviews using a topic guide with 6 open-ended questions.	Transcripts read by different members of research team. Independent coding by two authors. Codes analysed using framework analysis. Internal validation, consensus discussions.	Moderate. Some concerns for the lack of information on the researchers and their positionality.

### Meta-synthesis

The meta-synthesis resulted in two main themes: Circumstances and expectations; and Experiences of treatment (stage 3) with two and four (stage 2) descriptive themes, respectively. See Table 3 for certainty of evidence assessment and CERQual components grading for each descriptive theme.

**Table 3:** Main themes, descriptive themes, and confidence in the findings

Main theme (stage 3)	Descriptive theme (stage 2)	CERQual assessment	Component grading
Circumstances and expectations	Practical circumstances and social support were important for treatment to be feasible	Low	Methodological limitations: Moderate concerns Coherence: Very minor concerns Relevance: Minor concerns Adequacy of data: Minor concerns
	Expectations, previous experiences, and attitudes influenced how women experienced treatment.	Moderate	Methodological limitations: Moderate concerns Coherence: Very minor concerns Relevance: Very minor concerns Adequacy of data: Very minor concerns
Experiences of treatment	The received treatment’s modality was appreciated, but women had specific preferences concerning treatment intensity and individual adaptation	Moderate	Methodological limitations: Moderate concerns Coherence: Very minor concerns Relevance: Very minor concerns Adequacy of data: Very minor concerns
	The relationship with the clinician and perceptions about her/his competencies influenced how treatment was experienced	Moderate	Methodological limitations: Moderate concerns Coherence: Very minor concerns Relevance: Very minor concerns Adequacy of data: Very minor concerns
	Women expressed varying opinions about the treatments’ content, therapeutic approach, and the extent of their own expected contribution	Moderate	Methodological limitations: Moderate concerns Coherence: Very minor concerns Relevance: Very minor concerns Adequacy of data: Very minor concerns
	Women described positive treatment outcomes, but a few didn’t experience any improvement.	Moderate	Methodological limitations: Moderate concerns Coherence: Very minor concerns Relevance: Very minor concerns Adequacy of data: Very minor concerns

### Main theme 1: circumstances and expectations

*Practical circumstances and social support were important for treatment to be feasible*. Women in several studies described how important practical and social circumstances could be for them to take part in treatment.

Women talked about practical issues such as transportation [20] and childcare [19, 20] as fundamental. The internet-based therapies were appreciated for being accessible outside of office hours, despite some women having limited time for the program [22]. Another aspect was that many participants felt a lack of support from family and friends [20, 21, 26], and treatment was their only opportunity to talk about how they were feeling. Other women experienced some support from their family and meant that this support was vital for treatment.

*“I didn’t have anyone to talk to and no one actually knew about me being diagnosed with postnatal depression, my mum or anyone, no one knew, not even my partner. So it was quite nice just to offload on someone.” (HV listening visits* [26]*)*

*Expectations, previous experiences, and attitudes influenced how women experienced treatment.*

Women in most of the studies reported on how previous experiences, expectations, motivation, and beliefs about PPD influenced their experience of treatment. The women’s expectations of treatment were generally positive, however, there were those who didn’t believe that treatment would help them, grounded in a sense of hopelessness [21], or because of low confidence in health services, e.g., fear of not being understood [25, 26] or not being taken seriously [21]. Others talked about how their feelings of shame for being depressed, and thoughts about not being a good mother, affected how they believed treatment providers would perceive them [24, 26]. Obstacles to seeking help could also be previous negative experiences of certain health professionals [24, 25] or screening procedures [24], or fear of having their child removed if they revealed their depression [25].

*“None of us have ever admitted to having postnatal depression…there is still a stigma it’s incredible.” (Online-CBT* [21]*)*

There were women who had their own thoughts about why they were depressed, how it should be treated, and the potential of the treatments [19, 24, 25].

*“All you want is someone to actually listen to what you’re saying, even if it is complete crap and it’s all coming out wrong. You just want someone to say: “it’s alright, sit down and I’ll listen to what you’ve got to say”. That would do you the world of good and I think it would actually stop people from developing worse symptoms because people just won’t talk about it.” (HV person-centered intervention* [25]*)*

Some women worried that other participants in group sessions [20] or the health visitor [26] might disclose confidential information and chose therefore to not share all their thoughts and problems.

### Main theme 2: experiences of treatment

Overall, the included studies showed that the women were satisfied with the treatments they had received. Contributing factors were the format and content of the treatments, as well as the clinician’s approach.

*The received treatment’s modality was appreciated, but women had specific preferences concerning length, scope, and individual adaptations.*

Most of the studies included women’s thoughts and experiences of the treatment formats. Women who had received group therapies generally expressed positive experiences. They appreciated hearing other women’s stories, and that they could support each other [19, 20]. Objections towards the group format could be not feeling connected with others in the group, or that group therapy does not suit everyone [19]. Others would have liked more group sessions, and individual sessions as an adjunct to the group sessions [20].

Women who received home visits were satisfied to receive support in their own environment and with the continuity [23, 25, 26].

Some advantages mentioned by women who received internet-based CBT were accessibility and flexibility and to be able to work with the modules when they could fit it in [21, 22]. Internet therapy was experienced as less scrutinizing than face-to-face therapy [22], and less stigmatizing [21]. In one treatment model, the internet format was individualized with a personal e-mail from the therapist, which was appreciated [22].

*“When my maternal depression was really bad, there was no way I would have left my house to speak with a therapist — I was so weepy, shaky and terrified. …//… in those early weeks, the sort of anonymous nature of this program was a Godsend.” (Internet CBT* [22]*)*

In another study, where the internet format did not include any personal contact with the therapist, there were more dropouts, and the women had several suggestions for improvement, e.g., a more needs-based and relevant content, a more interactive format, and more individual support [21].

Regardless of treatment format, there were women who would have liked more treatment sessions and more flexibility and tailoring [20–22, 26]. Other women were happy with the number of sessions [22]. Ending therapy was described as a potentially anxiety provoking time [19, 26]. When women experienced continued support from family, other group participants, or professionals, this did not have to be a problem [19]. When no other support was available, however, ending therapy could be experienced negatively [26].

*“Just me thinking about it [the idea of no treatment after the visits] now makes me feel quite panicky… what would have been the point of ripping off the plaster and starting to abrade the wound, only to then just say, oh well.” (HV listening visits* [26]*)*

*The relationship with the clinician, and perceptions about her/his competencies influenced how treatment was experienced.*

Women in all eight studies talked about how they experienced their relationship to their health professional and their competencies.

The relationship with the nurse or therapist was described as important, regardless of treatment model or format. A good relationship was associated with trust and being able to talk about their depression. Some specific aspects of the relationship mentioned were chemistry [19, 22], credibility and broad competence [20, 21, 23, 26], e.g., knowledge of both infant’s needs and postnatal depression [23], interpersonal skills [20, 23], and intercultural and language competencies [20].

*She [health visitor] was so understanding and easy to talk to and willing to listen, that I actually opened up, otherwise I wouldn’t have done. (HV listening visits* [24]*)*

Sometimes, a good relationship was not established, or mothers did not feel confident that their therapist had the appropriate competence or necessary personal qualities [24–26], or was not flexible [26]. These experiences could lead women to decline further sessions [24, 25]. Some mothers wondered about who the home visitor’s primary interest was, the mother or the baby [25].

*Women expressed varying opinions about the treatments’ content, therapeutic approach, and the extent of their own expected contribution.*

Most studies included views concerning the specific content and therapeutic approach of the received treatment, and how this impacted the women’s own contribution.

Women who received home visits had many thoughts about the health visitors’ approach [23–26]. Active listening with an empathetic and non-judgmental approach was appreciated by many women as helpful for feelings of guilt and inadequacy [23].

Homework between sessions could be perceived as burdensome while also helpful [19, 20]. Some components were appreciated by many, for example, psychoeducation [22, 25], challenging thoughts [19] and storytelling in group sessions [20].

*We’ve analyzed all the reasons why I’ve been down and depressed, how to, sort of, challenge negative thoughts. (Individual CBT* [25] *)*

In the older studies there were women who didn’t find the home visits meaningful [24, 25], and these were sometimes described as too unstructured [24]. In the newer studies, however, the experiences of home visits were generally positive. Although the home visits were intended to be supportive, i.e., not giving advice, there were women who expressed a need for more clear and concrete advice from their home visitor [23, 25, 26].

Also, women who received CBT expressed positive experiences of their therapist’s personal approach [19, 22].

*[The internet therapist was] so helpful and thoughtful. She wasn’t hard on me like I am on myself and really made me stop and think about how I treat myself. (Individual CBT* [22]*)*

*Women described positive treatment outcomes, but a few did not experience any improvement.*

In general, women experienced their received intervention as helpful, and positive for their confidence and self-esteem. Treatment was described having led to a better understanding of their own distress and to insights about depression [20, 26], to acceptance and normalization, a generally more positive outlook on life and the future, and an increased sense of control [19, 20, 22].

*Not dwelling on all the negatives that I might feel, and she really made me see the little things that actually were big things that I’d done in life, so yeah, I think it made me a very different, you know, person. (Individual CBT* [19]*)*

A common experience following treatment was a better mother-infant relationship. Women described how they had gained knowledge about infants and about their own importance for their child’s development [23]. Many felt that their own improved mood had led to a better relationship with their child [19, 22] and that they had become more relaxed, patient, and secure in their parental role [22, 23].

*By 12 months, I felt I had the tools within myself to continue with sureness that I was a capable, confident mother. (Supportive home visits* [23]*)*

There were women who didn’t experience any improvement. In general, these women didn’t perceive supportive counselling as therapy [24], or as a sufficiently powerful intervention [26], and proceeded to seek other treatments instead. This was particularly notable in women with more chronic or recurrent depression [24, 26].

## Discussion

This meta-synthesis was based on studies that explored women’s experiences of CBT or supportive home visits. Treatments were individual or group-based.

Overall, the women were satisfied with their treatment, although various practical and social circumstances, as well as their own expectations, had an impact on their participation in and experience of treatment. Some findings reported were increased confidence and sense of control, and a better mother-infant relationship. Similarly, in an earlier meta-synthesis of psychological and psychosocial interventions for PPD, almost all included studies reported that women found their interventions helpful, specifically concerning their distress, their parenting, and their relationships [12].

Reoccurring themes in the current and previous syntheses were women’s wishes of being involved in decisions concerning their treatment and the impact of their own expectations of treatment [12, 14, 27]. They wanted to be involved in the choice of treatment type and format, and for treatments to be individualized, e.g., the selection and order of modules to be tailored to their personal preferences and practical circumstances. It has been argued that therapeutic alliance as well as flexibility, i.e., tailoring psychological treatments to the individual’s needs and circumstances can be more important than fidelity to treatment protocols [28]. In meta-analyses exploring the effectiveness of PPD, CBT has consistently demonstrated a favorable impact, e.g., Sockol et al. (2015) and Huang et al., (2018) [29, 30], with a relatively large number of studies confirming these results. Furthermore, this effect seems to be consistent for different formats (therapy delivered individually, in groups, or digitally) [31]. This is encouraging, suggesting that mothers’ preferences for various formats align with positive outcomes from an efficacy perspective, potentially instilling a sense of confidence in clinicians when considering the delivery of CBT in diverse forms. A recent synthesis investigating experiences of psychological treatment for depression in a broader context, excluding PPD [27], highlights how expectations concerning specific therapeutic approaches or formats can influence motivation and engagement in therapy.

The current synthesis identified some general expectations, e.g., positive previous experiences of care or expecting services to be under-resourced. There were also expectations, beliefs, and fears more specific to the perinatal period and related to being a new mother, in line with other syntheses in postpartum contexts [12, 14], such as motivation to get better, or fear of not being understood or not taken seriously. Mothers also worried they were, or would be seen as a bad mother, sometimes to the extent of fear of having their child removed. Our synthesis, as well as the one by Hadfield et al. [12] also identified women’s uncertainty concerning the health visitor’s role and competence to assess and support the mental wellbeing of mothers, which could sometimes lead to discontinuing treatment.

Women who had received group therapy expressed mainly positive experiences, consistent with McPherson et al.’s (2020) synthesis of non-postpartum treatments, where the group format contributed to normalization when realizing that they shared similar experiences and were not alone [27]. A negative aspect of the group format identified by McPherson et al., but less evident in our synthesis, was not feeling safe disclosing feelings, thus censoring what they shared. Common findings regarding CBT approaches were finding homework burdening, and more evident in McPherson’s synthesis than in the current, that CBT-modules could be difficult to apply.

Another finding, in line with Hadfield and Wittkowski [12] and a review by Daehn et al. investigating help-seeking among perinatal women [7], was the role of support from the partner or other family members to seek and take part in treatment. Practical circumstances such as transportation and childcare issues were evident for depressed mothers in the current and Hadfield’s synthesis, providing one reason for home visits being appreciated. However, the review of treatments in non-postpartum populations by McPherson et al. also found that transportation could be a problem and that remote therapy was preferred by some patients [27].

The significance of establishing a good relationship to their health professional was emphasized by the women, regardless of the treatment’s format or theoretical basis, consistent with other syntheses [12, 14, 27]. An empathetic, supportive, and non-judgmental approach was essential for the women’s wish to follow through with the treatment, and for their recovery. This is understandable considering how depression during this period is associated with feelings of anxiety, guilt, and worthlessness [32, 33]. In the synthesis by Megnin-Viggars et al. (2015) women emphasized continuity of care; for example, seeing the same nurse or therapist during the whole care period from assessment to treatment and follow-up, as important for being able to disclose symptoms of depression [14]. McPherson et al. (2020) emphasize patients’ descriptions of the therapeutic relationship as collaborative, and providing a space for sharing thoughts and feelings, and for receiving advice [27].

### Methodological strengths and limitations

Eight studies with low and moderate methodological limitations were included in the synthesis, and the findings concerning the women’s experiences were concordant among the included studies. Most studies had relatively few participants, but the interviews generated rich data with detailed descriptions of experiences. Most of the studies contributed data to all six descriptive themes, which were assessed as reflecting the variation in the findings, including contradicting and differing views and the complexity in the participants’ experiences. Authors had used semi-structured interview schedules with similar topic guides, likely explaining the similar types of narratives found. All studies lacked information about the researchers’ competencies and experience, and relationship to the participants; thus, how the authors’ preunderstandings were taken into consideration is largely unknown.

Other limitations are that the included studies were from the UK, Australia, and Canada and only one study targeted ethnic minorities, limiting the generalizability of our findings. Also, four of the eight included studies were more than 10 years old. Considering that we found some differences between the older versus newer studies in our review, it is possible that the delivery and formats of these treatments, mainly listening visits by a nurse or health visitor, may have changed over time suggesting a need for more updated studies.

A treatment with perhaps even better effect on depression during the perinatal period is Interpersonal therapy (IPT) [34], although less studied. It has been suggested that IPT may be especially suitable for women with postpartum depression because it focuses on improving relationships and addressing social support, which can be critical during the challenging postpartum period. IPT has been found to help women navigate the interpersonal challenges and changes that often accompany motherhood [34]. Unfortunately, our current meta-synthesis did not include any IPT studies, and limited data on treatment experiences are available. However, one study by Grote et al. (2009) reported high treatment satisfaction among mothers treated for PPD with IPT, as assessed through a brief questionnaire [35].

Strengths of the study include our following of an established method for synthesizing qualitative findings. Furthermore, and unlike previous meta-syntheses, we used CERQual to assess confidence in these findings.

## Conclusions

Most women described positive outcomes of the treatment they received, and findings suggested improved parent-related outcomes. The findings highlight the importance of involving women in decisions concerning treatment for postpartum depression so that support can be tailored to their circumstances and preferences. It is important for practitioners to take an interest in the women’s own thoughts about why they are depressed and their expectations of the treatment. Furthermore, the personal approach of the health professional; non-judgmental, sensitive, and able to convey hope is important during this vulnerable time. There is a need for updated research, including experiences of IPT.

### Electronic Supplementary Material

Below is the link to the electronic supplementary material.

Supplementary Material 1

## Data Availability

The search strategy and search terms used are available in Appendix 1. The data that support the findings of the current study is available in Swedish from the corresponding author upon reasonable request.
