# Supplementary material for: Women’s experiences of psychological treatment and psychosocial interventions for postpartum depression: a qualitative systematic review and meta-synthesis
Source: BMC Womens Health. 2023 Nov 14;23:604. doi: 10.1186/s12905-023-02772-8 (PMC10647124; doi:10.1186/s12905-023-02772-8)
Supplement: Supplementary file 1 — Supplementary Material 1 [file 12905_2023_2772_MOESM1_ESM.pdf]

Bilaga till rapport
Psykologisk behandling av postpartumdepression/Psychological Treatment of Postpartum Depression, rapport 358 (2022)

Bilaga 1 Sökstrategier/Appendix 1 Search strategies

Innehåll

Systematiska översikter/Systematic reviews 1
Psykologisk behandling av postpartumdepression/Psychological Treatment of Postpartum Depression 3
Upplevelser och erfarenheter av depressionsbehandling postpartum/Experiences of postpartum depression treatment 7
Hälsoekonomi/Health economics 20

Systematiska översikter/Systematic reviews

CINAHL via EBSCO 30 September 2021

Title: Postpartum depression- systematic reviews

Search terms		Items found
**Population: Postpartum depression**		
1.	(MH "Depression, Postpartum")	6,324
2.	TI ((perinatal or "peri natal" or peripart* or "peri part*" or postpart* or "post part*" or postnatal or "post natal") N3 (depress* or dysthymi* or melancholi*)) OR AB ((perinatal or "peri natal" or peripart* or "peri part*" or postpart* or "post part*" or postnatal or "post natal") N3 (depress* or dysthymi* or melancholi*)) OR SU ((perinatal or "peri natal" or peripart* or "peri part*" or postpart* or "post part*" or postnatal or "post natal") N3 (depress* or dysthymi* or melancholi*))	9,017
3.	*1-2 (OR)*	*9,017*
**Study types: systematic reviews, meta analysis**		
4.	MH "Systematic Review" OR ZT "systematic review" OR MH "Meta Analysis" OR ZT "meta analysis"	150,373
5.	(TI (systematic* n3 review*)) or (AB (systematic* n3 review*)) or (TI (systematic* n3 bibliographic*)) or (AB (systematic* n3 bibliographic*)) or (TI (systematic* n3 literature)) or (AB (systematic* n3 literature)) or (TI (comprehensive* n3 literature)) or (AB (comprehensive* n3 literature)) or (TI (comprehensive* n3 bibliographic*)) or (AB (comprehensive* n3 bibliographic*)) or (TI (integrative n3 review)) or (AB (integrative n3 review)) or (JN "Cochrane Database of Systematic Reviews") or (TI (information n2 synthesis)) or (TI (data n2 synthesis)) or (AB (information n2 synthesis)) or (AB (data n2 synthesis)) or (TI (data n2 extract*)) or (AB (data n2 extract*)) or (TI (medline or pubmed or psyclit or cinahl or (psycinfo not "psycinfo database") or "web of science" or scopus or embase)) or (AB (medline or pubmed or psyclit or cinahl or (psycinfo not "psycinfo database") or "web of science" or scopus or embase)) or (TI (meta-analy* or metaanaly*)) or (AB (meta-analy* or metaanaly*))	211,064
6.	*4-5 (OR)*	*243,934*
**Combined sets:**		
7.	3 AND 6	666
**Limits: publication year, language**		
8.	Limiters - Published Date: 20200101-20211231; Language: Danish, English, Norwegian, Swedish	
**Final result**		
**9.**	**7 AND 8**	**117**

The final search result, usually found at the end of the documentation, forms the list of abstracts.

AB = Abstract; **AU** = Author; **DE** = Term from the thesaurus; **MH**= Exact Subject Heading from CINAHL Subject Headings; **MM** = Major Concept; **TI** = Title; **TX** = All Text. Performs a keyword search of all the database's searchable fields; **ZC** = Methodology Index; ***** = Truncation; **" "** = Citation Marks; searches for an exact phrase; **N** = Near Operator (N) finds the words if they are a maximum of x words apart from one another, regardless of the order in which they appear.; **W** = Within Operator (W) finds the words if they are within x words of one another, in the order in which you entered them.

Embase via Elsevier 30 September 2021

Title: Postpartum depression – systematic reviews

Search terms		Items found
**Population: Postpartum depression**		
1.	'perinatal depression'/exp	14,332
2.	((perinatal OR 'peri natal' OR peripart* OR 'peri part*' OR postpart* OR 'post part*' OR postnatal OR 'post natal') NEAR/3 (depress* OR dysthymi* OR melanchol*)):ti,ab	11,945
3.	*1 OR 2*	*17,413*
**Study types: systematic reviews, meta analysis**		
4.	'systematic review'/de	314,013
5.	'meta analysis'/de	224,564
6.	[cochrane review]/lim	21,803
7.	((systematic* NEXT/3 (review* OR overview)):ti,ab) OR ((systematic* NEXT/3 bibliographic*):ti,ab) OR ((systematic* NEXT/3 literature):ti,ab) OR 'meta analy*':ti,ab OR metaanaly*:ti,ab	440,947
8.	*4-7 (OR)*	553,273
**Limits: publication year, language**		
9.	[2020-2021]/py	
10.	([danish]/lim OR [english]/lim OR [norwegian]/lim OR [swedish]/lim)	
**Combined sets**		
11.	*3 AND 8*	*1,153*
**Final result**		
**12.**	**9-11 (AND)**	**288**

The final search result, usually found at the end of the documentation, forms the list of abstracts.

/de = Term from the EMTREE controlled vocabulary; **/exp** = Includes terms found below this term in the EMTREE hierarchy **/mj** = Major Topic; **:ab** = Abstract; **:au** = Author; **:ti** = Article Title; **:ti,ab** = Title or abstract; ***** = Truncation; **' '** = Citation Marks; searches for an exact phrase; **NEAR/n** = Requests terms that are within 'n' words of each other in either direction; **NEXT/n** = Requests terms that are within 'n' words of each other in the order specified

Psykologisk behandling av postpartumdepression/Psychological Treatment of Postpartum Depression

CINAHL via EBSCO 20 Juni 2022

Title: Postpartum depression - therapy

Search terms		Items found
**Population: Postpartum depression**		
1.	(MH "Depression, Postpartum")	6,630
2.	TI (((postpart* or "post part*" or postnatal* or "post natal*" or peripart* or "peri part*" or perinatal or "peri natal" or postpregnan* or "post pregnan*" or maternal* or mother*) n5 (affective or depress* or distress* or "mood disorder*"))) OR AB (((postpart* or "post part*" or postnatal* or "post natal*" or peripart* or "peri part*" or perinatal or "peri natal" or postpregnan* or "post pregnan*" or maternal* or mother*) n5 (affective or depress* or distress* or "mood disorder*"))) OR SU (((postpart* or "post part*" or postnatal* or "post natal*" or peripart* or "peri part*" or perinatal or "peri natal" or postpregnan* or "post pregnan*" or maternal* or mother*) n5 (affective or depress* or distress* or "mood disorder*")))	14,599
3.	(MH "Postnatal Period")	11,114
4.	(MH "Depression+")	127,948
5.	S3 AND S4	1,343
6.	*S1 OR S2 OR S5*	*14,772*
**Intervention: Psychological therapies, counseling, support**		
7.	(MH "Psychotherapy+")	217,360
8.	(MH "Counseling+")	41,779
9.	(MH "Home Health Care")	24,988
10.	(MH "Home Visits") OR (MH "Psychiatric Home Care")	6,878
11.	(MH "Peer Group")	15,256
12.	TI ((abreaction or "acceptance and commitment" or ACT or "applied behav*" or CBT or consultation* or counsel* or "crisis intervention" or DBT or "emotion focused" or "free association" or hypnosis or hypnotherapy or intervent* or IPT or "life review" or "listening visit*" or logotherap* or mindfulness or "mom power" or "motivational interview*" or "peer group" or "peer support" or "problem solv*" or program* or psychoanaly* or "psycho-analy*" or psychodynamic* or "psycho dynamic*" or psychodrama or "psycho-drama*" or psychoeducat* or "psychologic desenzitization" or "psychological feedback" or psychosocial or psychotherap* or "psycho therap*" or "rational emotive" or "reminiscence" or "self control" or "self management" or "self system" or "socioenvironmental therapy" or support* or therap* or "transactional analysis" or train* or treat*)) OR AB ((abreaction or "acceptance and commitment" or ACT or "applied behav*" or CBT or consultation* or counsel* or "crisis intervention" or DBT or "emotion focused" or "free association" or hypnosis or hypnotherapy or intervent* or IPT or "life review" or "listening visit*" or logotherap* or mindfulness or "mom power" or "motivational interview*" or "peer group" or "peer support" or "problem solv*" or program* or psychoanaly* or "psycho-analy*" or psychodynamic* or "psycho dynamic*" or psychodrama or "psycho-drama*" or psychoeducat* or "psychologic desenzitization" or "psychological feedback" or psychosocial or psychotherap* or "psycho therap*" or "rational emotive" or "reminiscence" or "self control" or "self management" or "self system" or "socioenvironmental therapy" or support* or therap* or "transactional analysis" or train* or treat*)) OR SU ((abreaction or "acceptance and commitment" or ACT or "applied behav*" or CBT or consultation* or counsel* or "crisis intervention" or DBT or "emotion focused" or "free association" or hypnosis or hypnotherapy or intervent* or IPT or "life review" or "listening visit*" or logotherap* or mindfulness or "mom power" or "motivational interview*" or "peer group" or "peer support" or "problem solv*" or program* or psychoanaly* or "psycho-analy*" or psychodynamic* or "psycho dynamic*" or psychodrama or "psycho-drama*" or psychoeducat* or "psychologic desenzitization" or "psychological feedback" or psychosocial	3,867,103

	or psychotherap* or "psycho therap*" or "rational emotive" or "reminiscence" or "self control" or "self management" or "self system" or "socioenvironmental therapy" or support* or therap* or "transactional analysis" or train* or treat*))	
13.	TI ((behav* n3 (activat* or component? or contracting or defusion or guidance or management or modif* or rehab* or restructur* or technique* or train*))) OR AB ((behav* n3 (activat* or component? or contracting or defusion or guidance or management or modif* or rehab* or restructur* or technique* or train*))) OR SU ((behav* n3 (activat* or component? or contracting or defusion or guidance or management or modif* or rehab* or restructur* or technique* or train*)))	21,801
14.	TI (((cognitiv* or dialect*) n3 (behav* or component? or contracting or defusion or guidance or management or modif* or rehab* or restructur* or technique* or train*))) OR AB (((cognitiv* or dialect*) n3 (behav* or component? or contracting or defusion or guidance or management or modif* or rehab* or restructur* or technique* or train*))) OR SU (((cognitiv* or dialect*) n3 (behav* or component? or contracting or defusion or guidance or management or modif* or rehab* or restructur* or technique* or train*)))	33,280
15.	TI (((home or house) n3 (based or call* or care or service* or visit*))) OR AB (((home or house) n3 (based or call* or care or service* or visit*))) OR SU (((home or house) n3 (based or call* or care or service* or visit*)))	75,544
16.	(MH "Support, Psychosocial+")	96,482
17.	(MH "Self Care+")	57,682
18.	(MH "Self-Management")	2,108
19.	(MH "Support Groups+")	12,023
20.	TI (((self-care or selfcare or Self-help or selfhelp) n3 (administered or guided or instruct* or strateg* or supervised or tool*))) OR AB (((self-care or selfcare or Self-help or selfhelp) n3 (administered or guided or instruct* or strateg* or supervised or tool*))) OR SU (((self-care or selfcare or Self-help or selfhelp) n3 (administered or guided or instruct* or strateg* or supervised or tool*)))	1,658
21.	TI (((self-administered or selfadministered) n3 (guided or instruct* or strateg* or supervised))) OR AB (((self-administered or selfadministered) n3 (guided or instruct* or strateg* or supervised))) OR SU (((self-administered or selfadministered) n3 (guided or instruct* or strateg* or supervised)))	51
22.	(MH "Patient Education")	69,391
23.	*S7 OR S8 OR S9 OR S10 OR S11 OR S12 OR S13 OR S14 OR S15 OR S16 OR S17 OR S18 OR S19 OR S20 OR S21 OR S22*	*3,940,894*
**Intervention: Psychological therapies, counseling, support delivered via internet**		
24.	(MH "Computer Assisted Instruction")	8,228
25.	(MH "Therapy, Computer Assisted")	5,485
26.	(MH "Internet-Based Intervention")	437
27.	(MH "Telemedicine")	15,102
28.	(MH "Remote Consultation")	2,854
29.	(MH "Mobile Applications")	10,777
30.	(MH "Internet+")	164,120
31.	(MH "Cellular Phone+")	9,329
32.	(MH "Videoconferencing")	2,621
33.	(MH "Telecommunications")	2,609
34.	(MH "Computers, Hand-Held+")	8,230
35.	(MH "Medical Informatics")	5,433
36.	TI ((cybertherapy or "e-aid" or "e-counsel*" or "e-health program" or ehealth or "e-mental health" or "e-psychotherapy" or "e-psychology" or "e-therapy" or "guided self-help" or "online clinical work" or "self-help through the internet" or telecounseling or telepsychiatry or telepsychology or teletherapy or cCBT or c-CBT or "cyber-counseling" or "cyber-counsel*" or cybercounsel* or "digital health" or "e-consultation" or eCBT or e-CBT or econsultation or "e-counsel*" or ecounsel* or "e-health" or ehealth or emedicine or "e-medicine" or "emental health*" or "e-mental health*" or "e-portal" or eportal or epsych* or "e-psych*" or "e-therap*" or etherap* or "i-CBT" or ICBT or "m-health" or mhealth or "mobile health" or Deprexis or Interapy or WeChat*)) OR AB ((cybertherapy or "e-aid" or	16,148

	"e-counsel*" or "e-health program" or ehealth or "e-mental health" or "e-psychotherapy" or "e-psychology" or "e-therapy" or "guided self-help" or "online clinical work" or "self-help through the internet" or telecounseling or telepsychiatry or telepsychology or teletherapy or cCBT or c-CBT or "cyber-counseling" or "cyber-counsel*" or cybercounsel* or "digital health" or "e-consultation" or eCBT or e-CBT or econsultation or "e-counsel*" or ecounsel* or "e-health" or ehealth or emedicine or "e-medicine" or "emental health*" or "e-mental health*" or "e-portal" or eportal or epsych* or "e-psych*" or "e-therap*" or etherap* or "i-CBT" or ICBT or "m-health" or mhealth or "mobile health" or Deprexis or Interapy or WeChat*)) OR SU ((cybertherapy or "e-aid" or "e-counsel*" or "e-health program" or ehealth or "e-mental health" or "e-psychotherapy" or "e-psychology" or "e-therapy" or "guided self-help" or "online clinical work" or "self-help through the internet" or telecounseling or telepsychiatry or telepsychology or teletherapy or cCBT or c-CBT or "cyber-counseling" or "cyber-counsel*" or cybercounsel* or "digital health" or "e-consultation" or eCBT or e-CBT or econsultation or "e-counsel*" or ecounsel* or "e-health" or ehealth or emedicine or "e-medicine" or "emental health*" or "e-mental health*" or "e-portal" or eportal or epsych* or "e-psych*" or "e-therap*" or etherap* or "i-CBT" or ICBT or "m-health" or mhealth or "mobile health" or Deprexis or Interapy or WeChat*))	
37.	S24 OR S25 OR S26 OR S27 OR S28 OR S29 OR S30 OR S31 OR S32 OR S33 OR S34 OR S35 OR S36	225,184
**Study types: Study types: randomised controlled trials and other trials edited and based on Glanville et al. 2019 [1]**		
38.	MH "Randomized Controlled Trials+" OR MH "Double-Blind Studies" OR MH "Single-Blind Studies" OR MH "Random Assignment" MH "Pretest-Posttest Design" OR MH "Cluster Sample" OR MH "Sample Size" OR MH "Crossover Design" OR MH "Comparative Studies" OR MH "Placebos" OR PT (Randomized Controlled Trial) OR MH "Therapeutic Trials" OR MH "Triple-Blind Studies" OR MH "Clinical Trials" OR MH "Intervention Trials" OR MH "Preventive Trials" OR PT (Randomized Controlled Trial) OR TI (randomised OR randomized OR phase 3 OR phase3 OR phase III) OR AB (random* OR phase 3 OR phase3 OR phase III) OR TI (trial) OR AB (assigned OR allocated OR control) OR AB (control W5 group) OR AB (cluster W3 rct)	1,317,785
**Combined sets:**		
39.	6 AND (23 OR 37) AND 38	2,926
**Final result**		
**40.**	**39**<br>**Limiters - Language: Danish, English, Norwegian, Swedish**	**2,876**

The final search result, usually found at the end of the documentation, forms the list of abstracts.

AB = Abstract; **AU** = Author; **DE** = Term from the thesaurus; **MH**= Exact Subject Heading from CINAHL Subject Headings; **MM** = Major Concept; **TI** = Title; **TX** = All Text. Performs a keyword search of all the database's searchable fields; **ZC** = Methodology Index; ***** = Truncation; **" "** = Citation Marks; searches for an exact phrase; **N** = Near Operator (N) finds the words if they are a maximum of x words apart from one another, regardless of the order in which they appear.; **W** = Within Operator (W) finds the words if they are within x words of one another, in the order in which you entered them.

Cochrane Library via Wiley 20 Juni 2022

Title: Postpartum depression - therapy

	Search terms	Items found
**Population: Postpartum depression**		
1.	MeSH descriptor: [Depression, Postpartum] explode all trees	706
2.	MeSH descriptor: [Postpartum Period] explode all trees	1885
3.	MeSH descriptor: [Depressive Disorder] explode all trees	13291
4.	(((postpart* or "post part*" or postnatal* or "post natal*" or peripart* or "peri part*" or perinatal or "peri natal" or postpregnan* or "post pregnan*" or maternal* or mother*)	3911

[1] Glanville J, Dooley G, Wisniewski S, Foxlee R, Noel-Storr A. Development of search filter to identify reports of controlled clinical trials within CINAHLPlus. Health Info Libr J. 2019 Mar;36(1):73-90. doi: 10.1111/hir.12251. Epub 2019Feb 8. PubMed PMID: 30737884.

	NEAR/5 (affective or depress* or distress* or "mood disorder*"))):ti,ab,kw (Word variations have been searched)	
5.	2 AND 3	85
**Final result**		
**6.**	**1 OR 4 OR 5**	**CENTRAL/ 3806**

The final search result, usually found at the end of the documentation, forms the list of abstracts.

:au = Author; MeSH = Term from the Medline controlled vocabulary, including terms found below this term in the MeSH hierarchy; **this term only** = Does not include terms found below this term in the MeSH hierarchy; **:ti** = Title; **:ab** = Abstract; **:kw** = Keyword; ***** = Truncation; **" "** = Citation Marks; searches for an exact phrase; **CDSR** = Cochrane Database of Systematic Review; **Cochrane Protocols** = Protocols of systematic reviews registered in Cochrane Library; **CENTRAL** = Cochrane Central Register of Controlled Trials, "trials"

Upplevelser och erfarenheter av depressionsbehandling postpartum/Experiences of postpartum depression treatment

CINAHL via EBSCO 27 June 2022

Title: Postpartum depression – patients experiences of care (qualitative studies)

Search terms		Items found
**Population: Postpartum depression**		
1.	(MH "Depression, Postpartum")	6,643
2.	TI (((postpart* or "post part*" or postnatal* or "post natal*" or peripart* or "peri part*" or perinatal or "peri natal" or postpregnan* or "post pregnan*" or maternal* or mother*) n5 (affective or depress* or distress* or "mood disorder*"))) OR AB (((postpart* or "post part*" or postnatal* or "post natal*" or peripart* or "peri part*" or perinatal or "peri natal" or postpregnan* or "post pregnan*" or maternal* or mother*) n5 (affective or depress* or distress* or "mood disorder*"))) OR SU (((postpart* or "post part*" or postnatal* or "post natal*" or peripart* or "peri part*" or perinatal or "peri natal" or postpregnan* or "post pregnan*" or maternal* or mother*) n5 (affective or depress* or distress* or "mood disorder*")))	14,611
3.	(MH "Postnatal Period")	11,131
4.	(MH "Depression+")	128,146
5.	S3 AND S4	1,345
6.	*S1 OR S2 OR S5*	*14,785*
**Intervention: Psychological therapies, counseling, support**		
7.	(MH "Psychotherapy+") OR (MH "Counseling+") OR (MH "Home Health Care") OR (MH "Home Visits") OR (MH "Psychiatric Home Care") OR (MH "Peer Group") OR (MH "Support, Psychosocial+") OR (MH "Self Care+") OR (MH "Self-Management") OR (MH "Support Groups+") OR (MH "Patient Education")	394,010
8.	TI ((abreaction or "acceptance and commitment" or ACT or "applied behav*" or CBT or consultation* or counsel* or "crisis intervention" or DBT or "emotion focused" or "free association" or hypnosis or hypnotherapy or intervent* or IPT or "life review" or "listening visit*" or logotherap* or mindfulness or "mom power" or "motivational interview*" or "peer group" or "peer support" or "problem solv*" or program* or psychoanaly* or "psycho-analy*" or psychodynamic* or "psycho dynamic*" or psychodrama or "psycho-drama*" or psychoeducat* or "psychologic desenzitization" or "psychological feedback" or psychosocial or psychotherap* or "psycho therap*" or "rational emotive" or "reminiscence" or "self control" or "self management" or "self system" or "socioenvironmental therapy" or support* or therap* or "transactional analysis" or train* or treat*)) OR AB ((abreaction or "acceptance and commitment" or ACT or "applied behav*" or CBT or consultation* or counsel* or "crisis intervention" or DBT or "emotion focused" or "free association" or hypnosis or hypnotherapy or intervent* or IPT or "life review" or "listening visit*" or logotherap* or mindfulness or "mom power" or "motivational interview*" or "peer group" or "peer support" or "problem solv*" or program* or psychoanaly* or "psycho-analy*" or psychodynamic* or "psycho dynamic*" or psychodrama or "psycho-drama*" or psychoeducat* or "psychologic desenzitization" or "psychological feedback" or psychosocial or psychotherap* or "psycho therap*" or "rational emotive" or "reminiscence" or "self control" or "self management" or "self system" or "socioenvironmental therapy" or support* or therap* or "transactional analysis" or train* or treat*)) OR SU ((abreaction or "acceptance and commitment" or ACT or "applied behav*" or CBT or consultation* or counsel* or "crisis intervention" or DBT or "emotion focused" or "free association" or hypnosis or hypnotherapy or intervent* or IPT or "life review" or "listening visit*" or logotherap* or mindfulness or "mom power" or "motivational interview*" or "peer group" or "peer support" or "problem solv*" or program* or psychoanaly* or "psycho-analy*" or psychodynamic* or "psycho dynamic*" or psychodrama or "psycho-drama*" or psychoeducat* or "psychologic desenzitization" or "psychological feedback" or psychosocial or psychotherap* or "psycho therap*" or "rational emotive" or "reminiscence" or "self	3,871,234

	control" or "self management" or "self system" or "socioenvironmental therapy" or support* or therap* or "transactional analysis" or train* or treat*))	
9.	TI ((behav* n3 (activat* or component? or contracting or defusion or guidance or management or modif* or rehab* or restructur* or technique* or train*))) OR AB ((behav* n3 (activat* or component? or contracting or defusion or guidance or management or modif* or rehab* or restructur* or technique* or train*))) OR SU ((behav* n3 (activat* or component? or contracting or defusion or guidance or management or modif* or rehab* or restructur* or technique* or train*)))	21,834
10.	TI (((cognitiv* or dialect*) n3 (behav* or component? or contracting or defusion or guidance or management or modif* or rehab* or restructur* or technique* or train*))) OR AB (((cognitiv* or dialect*) n3 (behav* or component? or contracting or defusion or guidance or management or modif* or rehab* or restructur* or technique* or train*))) OR SU (((cognitiv* or dialect*) n3 (behav* or component? or contracting or defusion or guidance or management or modif* or rehab* or restructur* or technique* or train*)))	33,329
11.	TI (((home or house) n3 (based or call* or care or service* or visit*))) OR AB (((home or house) n3 (based or call* or care or service* or visit*))) OR SU (((home or house) n3 (based or call* or care or service* or visit*)))	75,613
12.	TI (((self-care or selfcare or Self-help or selfhelp) n3 (administered or guided or instruct* or strateg* or supervised or tool*))) OR AB (((self-care or selfcare or Self-help or selfhelp) n3 (administered or guided or instruct* or strateg* or supervised or tool*))) OR SU (((self-care or selfcare or Self-help or selfhelp) n3 (administered or guided or instruct* or strateg* or supervised or tool*)))	1,664
13.	TI (((self-administered or selfadministered) n3 (guided or instruct* or strateg* or supervised))) OR AB (((self-administered or selfadministered) n3 (guided or instruct* or strateg* or supervised))) OR SU (((self-administered or selfadministered) n3 (guided or instruct* or strateg* or supervised)))	51
14.	*S7 OR S8 OR S9 OR S10 OR S11 OR S12 OR S13*	3,945,073
**Intervention: Psychological therapies, counseling, support delivered via internet**		
15.	(MH "Computer Assisted Instruction") OR (MH "Therapy, Computer Assisted") OR (MH "Internet-Based Intervention") OR (MH "Telemedicine") OR (MH "Remote Consultation") OR (MH "Mobile Applications") OR (MH "Internet+") OR (MH "Cellular Phone+") OR (MH "Videoconferencing") OR (MH "Telecommunications") OR (MH "Computers, Hand-Held+") OR (MH "Medical Informatics")	215,531
16.	TI ((cybertherapy or "e-aid" or "e-counsel*" or "e-health program" or ehealth or "e-mental health" or "e-psychotherapy" or "e-psychology" or "e-therapy" or "guided self-help" or "online clinical work" or "self-help through the internet" or telecounseling or telepsychiatry or telepsychology or teletherapy or cCBT or c-CBT or "cyber-counseling" or "cyber-counsel*" or cybercounsel* or "digital health" or "e-consultation" or eCBT or e-CBT or econsultation or "e-counsel*" or ecounsel* or "e-health" or ehealth or emedicine or "e-medicine" or "emental health*" or "e-mental health*" or "e-portal" or eportal or epsych* or "e-psych*" or "e-therap*" or etherap* or "i-CBT" or ICBT or "m-health" or mhealth or "mobile health" or Deprexis or Interapy or WeChat*)) OR AB ((cybertherapy or "e-aid" or "e-counsel*" or "e-health program" or ehealth or "e-mental health" or "e-psychotherapy" or "e-psychology" or "e-therapy" or "guided self-help" or "online clinical work" or "self-help through the internet" or telecounseling or telepsychiatry or telepsychology or teletherapy or cCBT or c-CBT or "cyber-counseling" or "cyber-counsel*" or cybercounsel* or "digital health" or "e-consultation" or eCBT or e-CBT or econsultation or "e-counsel*" or ecounsel* or "e-health" or ehealth or emedicine or "e-medicine" or "emental health*" or "e-mental health*" or "e-portal" or eportal or epsych* or "e-psych*" or "e-therap*" or etherap* or "i-CBT" or ICBT or "m-health" or mhealth or "mobile health" or Deprexis or Interapy or WeChat*)) OR SU ((cybertherapy or "e-aid" or "e-counsel*" or "e-health program" or ehealth or "e-mental health" or "e-psychotherapy" or "e-psychology" or "e-therapy" or "guided self-help" or "online clinical work" or "self-help through the internet" or telecounseling or telepsychiatry or telepsychology or teletherapy or cCBT or c-CBT or "cyber-counseling" or "cyber-counsel*" or cybercounsel* or "digital health" or "e-consultation" or eCBT or e-CBT or econsultation or "e-counsel*" or ecounsel* or "e-health" or ehealth or emedicine or "e-medicine" or "emental health*" or "e-mental health*" or "e-	16,237

	portal" or eportal or epsych* or "e-psych*" or "e-therap*" or etherap* or "i-CBT" or ICBT or "m-health" or mhealth or "mobile health" or Deprexis or Interapy or WeChat*))	
17.	S15 OR S16	225,552
**Evaluation: Patients experiences of care**		
18.	(MH "Attitude to Medical Treatment") OR (MH "Attitude to Health") OR (MH "Consumer Attitudes") OR (MH "Health Beliefs") OR (MH "Patient Satisfaction+") OR (MH "Patient Attitudes") OR (MH "Personal Satisfaction") OR (MH "Patient Preference") OR (MH "Attitude") OR (MH "Consumer Satisfaction") OR (MH "Patients/PF") OR (MH "Outpatients/PF") OR (MH "Maternal Attitudes")<br>(MH "Mothers/PF") OR (MH "Adolescent Mothers/PF") OR (MH "Expectant Mothers/PF") OR (MH "Mothers, Working/PF") OR (MH "Multiparas/PF") OR (MH "Primiparas/PF") OR (MH "Surrogate Mothers/PF")	217,634
19.	((client* or patient* or women* or mother* or matern* or mum* or outpatient*) N15 (accept* or attitude* or belief* or believ* or disbelief* or disbeliev* or dissatisf* or encounter* or expectation* or experience* or improvement* or interpret* or involvement or meaning or percept* or perspective* or satisf* or stigma* or relation or "self-report" or "treatment barrier*" or trust* or understanding* or unsatisf* or value or view*))	674,841
20.	S18 OR S19	758,107
21.	TI ((postpart* or "post part*" or postnatal* or "post natal*" or peripart* or "peri part*" or perinatal or "peri natal" or postpregnan* or "post pregnan*") N5 (affective or depress* or distress* or "mood disorder*")) N15 (accept* or attitude* or belief* or believ* or disbelief* or disbeliev* or dissatisf* or encounter* or expectation* or experience* or improvement* or interpret* or involvement or meaning or percept* or perspective* or satisf* or stigma* or relation or "self-report" or "treatment barrier*" or trust* or understanding* or unsatisf* or value or view*) OR AB ((postpart* or "post part*" or postnatal* or "post natal*" or peripart* or "peri part*" or perinatal or "peri natal" or postpregnan* or "post pregnan*") N5 (affective or depress* or distress* or "mood disorder*")) N15 (accept* or attitude* or belief* or believ* or disbelief* or disbeliev* or dissatisf* or encounter* or expectation* or experience* or improvement* or interpret* or involvement or meaning or percept* or perspective* or satisf* or stigma* or relation or "self-report" or "treatment barrier*" or trust* or understanding* or unsatisf* or value or view*) OR SU ((postpart* or "post part*" or postnatal* or "post natal*" or peripart* or "peri part*" or perinatal or "peri natal" or postpregnan* or "post pregnan*") N5 (affective or depress* or distress* or "mood disorder*")) N15 (accept* or attitude* or belief* or believ* or disbelief* or disbeliev* or dissatisf* or encounter* or expectation* or experience* or improvement* or interpret* or involvement or meaning or percept* or perspective* or satisf* or stigma* or relation or "self-report" or "treatment barrier*" or trust* or understanding* or unsatisf* or value or view*)	1,778
**Study types: qualitative study design**		
22.	(MH "Qualitative Studies+") OR (MH "Empirical Research") OR (MH "Questionnaires+") OR (MH "Videorecording") OR (MH "Research, Nursing") OR (MH "Research, Midwifery") OR (MH "Survey Research") OR (MH "Phenomenological Research") OR (MH "Grounded Theory") OR (MH "Multimethod Studies") OR (MH "Content Analysis") OR (MH "Constant Comparative Method") OR (MH "Thematic Analysis") OR (MH "Audiorecording") OR (MH "Focus Groups") OR (MH "Interviews+") OR (MH "Narratives+") OR (MH "Self Report+") OR (MH "Surveys+") OR (MH "Field Studies") OR (MH "Cluster Sample+") OR (MH "Comparative Studies") OR (MH "Life Experiences") OR (MH "Ethnology") OR (MH "Ethnonursing Research") OR (MH "Ethnological Research") OR (MH "Ethnographic Research") OR (MH "Phenomenology") OR (MH "Triangulation") OR (MH "Storytelling")	1,371,687
23.	TI (("action research" or "cluster sample*" or "constant comparative method*" or "key informant*" or "maximum variation sampling*" or qualitative* or interview* or "focus group*" or phenomeno* or ethnolog* or ethnograph* or ethnonurs* or ethnomethodolog* or "meta-ethnograph*" or hermeneutic* or "grounded theory" or observation or "lived experience*" or narrat* or "content analys*" or "purposive sample*" or "thematic analys*" or "theoretical sample*" or "triangulation design")) OR AB (("action research" or "cluster sample*" or "constant comparative method*" or "key informant*" or "maximum variation sampling*" or qualitative* or interview* or "focus group*" or phenomeno* or ethnolog* or ethnograph* or ethnonurs* or ethnomethodolog* or "meta-ethnograph*" or hermeneutic*	675,836

	or "grounded theory" or observation or "lived experience*" or narrat* or "content analys*" or "purposive sample*" or "thematic analys*" or "theoretical sample*" or "triangulation design")) OR SU (("action research" or "cluster sample*" or "constant comparative method*" or "key informant*" or "maximum variation sampling*" or qualitative* or interview* or "focus group*" or phenomeno* or ethnolog* or ethnograph* or ethnonurs* or ethnomethodolog* or "meta-ethnograph*" or hermeneutic* or "grounded theory" or observation or "lived experience*" or narrat* or "content analys*" or "purposive sample*" or "thematic analys*" or "theoretical sample*" or "triangulation design"))	
24.	TI ((((field n2 (research or study or studies or work))) OR ((grounded n2 (theor* or study or studies or research or analys*))) OR (((lived or life) n2 (experience* or story or stories))) OR (((video or tape) n2 record*)) OR ((("semi-structured" or semistructured or unstructured or informal or "in-depth" or indepth or "face-to-face" or structured or guide) n3 (interview* or discussion* or open ended or questionnaire*)))) OR AB ((((field n2 (research or study or studies or work))) OR ((grounded n2 (theor* or study or studies or research or analys*))) OR (((lived or life) n2 (experience* or story or stories))) OR (((video or tape) n2 record*)) OR ((("semi-structured" or semistructured or unstructured or informal or "in-depth" or indepth or "face-to-face" or structured or guide) n3 (interview* or discussion* or open ended or questionnaire*)))) OR SU ((((field n2 (research or study or studies or work))) OR ((grounded n2 (theor* or study or studies or research or analys*))) OR (((lived or life) n2 (experience* or story or stories))) OR (((video or tape) n2 record*)) OR ((("semi-structured" or semistructured or unstructured or informal or "in-depth" or indepth or "face-to-face" or structured or guide) n3 (interview* or discussion* or open ended or questionnaire*))))	235,105
25.	(mixed or multi) N1 (method* or stud*)	33,133
26.	S22 OR S23 OR S24 OR S25	1,628,272
**Combined sets:**		
27.	*((6 AND 20) OR 21)*	*5,988*
28.	*(14 OR 17) AND 26 AND 27*	*3,844*
**Final result**		
**29.**	**28**<br>**Limiters - Language: Danish, English, Norwegian, Swedish**	**3,772**

The final search result, usually found at the end of the documentation, forms the list of abstracts.

AB = Abstract; **AU** = Author; **DE** = Term from the thesaurus; **MH**= Exact Subject Heading from CINAHL Subject Headings; **MM** = Major Concept; **TI** = Title; **TX** = All Text. Performs a keyword search of all the database's searchable fields; **ZC** = Methodology Index; ***** = Truncation; **" "** = Citation Marks; searches for an exact phrase; **N** = Near Operator (N) finds the words if they are a maximum of x words apart from one another, regardless of the order in which they appear.; **W** = Within Operator (W) finds the words if they are within x words of one another, in the order in which you entered them.

Cochrane Library via Wiley 27 June 2022 (CENTRAL)

Title: Postpartum depression – patients experiences of care (qualitative studies)

Search terms		Items found
**Population: Postpartum depression**		
1.	MeSH descriptor: [Depression, Postpartum] explode all trees	706
2.	MeSH descriptor: [Postpartum Period] explode all trees	1885
3.	MeSH descriptor: [Depressive Disorder] explode all trees	13291
4.	(((postpart* or "post part*" or postnatal* or "post natal*" or peripart* or "peri part*" or perinatal or "peri natal" or postpregnan* or "post pregnan*" or maternal* or mother*) NEAR/5 (affective or depress* or distress* or "mood disorder*"))):ti,ab,kw (Word variations have been searched)	3911
5.	2 AND 3	85
**Final result**		
**6.**	**1 OR 4 OR 5**	**CENTRAL/ 3806**

Embase via Elsevier 27 June 2022

Title: Postpartum depression – patients experiences of care (qualitative studies)

Search terms		Items found
**Population: Postpartum depression**		
1.	'perinatal depression'/de OR 'postnatal depression'/exp	14,746
2.	'puerperium'/de AND 'depression'/de	1,806
3.	((postpart* OR 'post part*' OR postnatal* OR 'post natal*' OR peripart* OR 'peri part*' OR perinatal OR 'peri natal' OR postpregnan* OR 'post pregnan*' OR maternal* OR mother*) NEAR/3 (affective OR depress* OR distress* OR 'mood disorder*')):ti,ab	21,844
4.	*1 OR 2 OR 3*	*26,308*
**Intervention: Psychological therapies, counseling, support**		
5.	'psychotherapy'/exp OR 'counseling'/de OR 'directive counseling'/de OR 'e-counseling'/de OR 'family counseling'/de OR 'motivational interviewing'/de OR 'parent counseling'/de OR 'patient counseling'/de OR 'patient guidance'/de OR 'psychological counseling'/de OR 'home care'/de OR 'home mental health care'/de OR 'home visit'/de OR 'social support'/exp OR 'peer counseling'/de OR 'psychological care'/exp	607,092
6.	abreaction:ti,kw,ab OR 'acceptance and commitment':ti,kw,ab OR act:ti,kw,ab OR 'applied behav*':ti,kw,ab OR cbt:ti,kw,ab OR consultation*:ti,kw,ab OR counsel*:ti,kw,ab OR 'crisis intervention':ti,kw,ab OR dbt:ti,kw,ab OR 'emotion focused':ti,kw,ab OR 'free association':ti,kw,ab OR hypnosis:ti,kw,ab OR hypnotherapy:ti,kw,ab OR intervent*:ti,kw,ab OR ipt:ti,kw,ab OR 'life review':ti,kw,ab OR 'listening visit*':ti,kw,ab OR logotherap*:ti,kw,ab OR mindfulness:ti,kw,ab OR 'mom power':ti,kw,ab OR 'motivational interview*':ti,kw,ab OR 'peer group':ti,kw,ab OR 'peer support':ti,kw,ab OR 'problem solv*':ti,kw,ab OR program*:ti,kw,ab OR psychoanaly*:ti,kw,ab OR 'psycho-analy*':ti,kw,ab OR psychodynamic*:ti,kw,ab OR 'psycho dynamic*':ti,kw,ab OR psychodrama:ti,kw,ab OR 'psycho-drama*':ti,kw,ab OR psychoeducat*:ti,kw,ab OR 'psychologic desenzitization':ti,kw,ab OR 'psychological feedback':ti,kw,ab OR psychosocial:ti,kw,ab OR psychotherap*:ti,kw,ab OR 'psycho therap*':ti,kw,ab OR 'rational emotive':ti,kw,ab OR 'reminiscence':ti,kw,ab OR 'self control':ti,kw,ab OR 'self management':ti,kw,ab OR 'self system':ti,kw,ab OR 'socioenvironmental therapy':ti,kw,ab OR support*:ti,kw,ab OR therap*:ti,kw,ab OR 'transactional analysis':ti,kw,ab OR train*:ti,kw,ab OR treat*:ti,kw,ab	14,543,977
7.	(behav* NEAR/3 (activat* OR component? OR contracting OR defusion OR guidance OR management OR modif* OR rehab* OR restructur* OR technique* OR train*)):ti,ab,kw	54,924
8.	(cognitiv* OR dialect*) NEAR/3 (behav* OR component* OR contracting OR defusion OR guidance OR management OR modif* OR rehab* OR restructur* OR technique* OR train*)	112,166
9.	((home OR house) NEAR/3 (based OR call* OR care OR service* OR visit*)):ti,ab,kw	82,813
10.	(('self care' OR selfcare OR 'self help' OR selfhelp) NEAR/3 (administered OR guided OR instruct* OR strateg* OR supervised OR tool*)):ti,ab,kw	2,501
11.	(('self administered' OR selfadministered) NEAR/3 (guided OR instruct* OR strateg* OR supervised)):ti,ab,kw	151
12.	'self help'/de OR 'patient education'/exp OR 'self care'/de	193.370
13.	*5 OR 6 OR 7 OR 8 OR 9 OR 10 OR 11 OR 12*	*14,800,103*
**Intervention: Psychological therapies, counseling, support delivered via internet**		
14.	'internet'/de OR 'medical informatics'/de OR 'telemedicine'/de OR 'teleconsultation'/exp OR 'telepsychiatry'/de OR 'telepsychology'/de OR 'video consultation'/de OR 'mobile application'/exp OR 'mobile phone'/exp OR 'videoconferencing'/exp OR 'telecommunication'/de OR 'personal digital assistant'/exp OR 'computer'/de	341,806
15.	cybertherapy:ti,ab OR 'e aid':ti,ab OR 'e counseling':ti,ab OR ecounseling:ti,ab OR 'e-health program':ti,ab OR 'e-mental health':ti,ab OR 'e psychotherapy':ti,ab OR 'e psychology':ti,ab OR 'e therapy':ti,ab OR 'guided self-help':ti,ab OR 'online clinical work':ti,ab OR 'self-help through the internet':ti,ab OR telecounseling:ti,ab OR telepsychiatry:ti,ab OR telepsychology:ti,ab OR teletherapy:ti,ab OR ccbt:ti,ab OR 'c	27,603

	cbt':ti,ab OR 'cyber-counseling':ti,ab OR 'cyber-counsel*':ti,ab OR cybercounsel*:ti,ab OR 'digital health':ti,ab OR 'e consultation':ti,ab OR ecbt:ti,ab OR 'e cbt':ti,ab OR econsultation:ti,ab OR 'e counsel*':ti,ab OR ecounsel*:ti,ab OR 'e health':ti,ab OR ehealth:ti,ab OR emedicine:ti,ab OR 'e medicine':ti,ab OR 'emental health*':ti,ab OR 'e-mental health*':ti,ab OR 'e portal':ti,ab OR eportal:ti,ab OR epsych*:ti,ab OR 'e psych*':ti,ab OR 'e therap*':ti,ab OR etherap*:ti,ab OR 'i cbt':ti,ab OR icbt:ti,ab OR 'm health':ti,ab OR mhealth:ti,ab OR 'mobile health':ti,ab OR deprexis:ti,ab OR interapy:ti,ab OR wechat*:ti,ab	
16.	*14 OR 15*	*355,990*
**Evaluation: Patients experiences of care**		
17.	'patient attitude' OR 'patient preference'/exp/mj OR 'patient satisfaction'/de OR 'experience'/exp	268,407
18.	((client* OR patient* OR women* OR mother* OR matern* OR mum* OR outpatient*) NEAR/15 (accept* OR attitude* OR belief* OR believ* OR disbelief* OR disbeliev* OR dissatisf* OR encounter* OR expectation* OR experience* OR improvement* OR interpret* OR involvement OR meaning OR percept* OR perspective* OR satisf* OR stigma* OR relation OR 'self-report' OR 'treatment barrier*' OR trust* OR understanding* OR unsatisf* OR value OR view*)):ti,ab	2,213,298
19.	((postpart* OR 'post part*' OR postnatal* OR 'post natal*' OR peripart* OR 'peri part*' OR perinatal OR 'peri natal' OR postpregnan* OR 'post pregnan*') NEAR/5 (affective OR depress* OR distress* OR 'mood disorder*') NEAR/15 (accept* OR attitude* OR belief* OR believ* OR disbelief* OR disbeliev* OR dissatisf* OR encounter* OR expectation* OR experience* OR improvement* OR interpret* OR involvement OR meaning OR percept* OR perspective* OR satisf* OR stigma* OR relation OR 'self-report' OR 'treatment barrier*' OR trust* OR understanding* OR unsatisf* OR value OR view*)):ti,ab	2,941
20.	*17 OR 18*	*2,345,538*
**Study types: qualitative study design**		
21.	'empirical research'/exp OR 'questionnaire'/exp OR 'nursing research'/exp OR 'phenomenology'/exp OR 'survey methodology'/exp OR 'grounded theory'/exp OR 'content analysis'/exp OR 'multimethod study'/exp OR 'constant comparative method'/exp OR 'discourse analysis'/exp OR 'thematic analysis'/exp OR 'self report'/exp OR 'narrative'/exp OR 'interview'/exp OR 'comparative study'/de OR 'field study'/exp OR 'ethnology'/exp OR 'cluster analysis'/exp OR 'storytelling'/exp OR 'personal experience'/exp OR 'recording'/exp	2,519,833
22.	(grounded NEAR/2 (theor* OR study OR studies OR research OR analys*)):ti,ab	18,588
23.	((lived OR life) NEAR/2 (experience* OR story OR stories)):ti,ab	27,110
24.	((video OR tape) NEAR/2 record*):ti,ab	21,883
25.	(('semi-structured' OR semistructured OR unstructured OR informal OR 'in-depth' OR indepth OR 'face-to-face' OR structured OR guide) NEAR/3 (interview* OR discussion* OR 'open ended' OR questionnaire*)):ti,ab	201,585
26.	'action research':ti,ab OR 'cluster sample*':ti,ab OR 'constant comparative method*':ti,ab OR 'key informant*':ti,ab OR 'maximum variation sampling*':ti,ab OR qualitative*:ti,ab OR interview*:ti,ab OR 'focus group*':ti,ab OR phenomeno*:ti,ab OR ethnolog*:ti,ab OR ethnograph*:ti,ab OR ethnonurs*:ti,ab OR ethnomethodolog*:ti,ab OR 'meta-ethnograph*':ti,ab OR hermeneutic*:ti,ab OR 'grounded theory':ti,ab OR observation:ti,ab OR 'lived experience*':ti,ab OR narrat*:ti,ab OR 'content analys*':ti,ab OR 'purposive sample*':ti,ab OR 'thematic analys*':ti,ab OR 'theoretical sample*':ti,ab OR 'triangulation design':ti,ab	1,633,585
27.	(field NEAR/2 (research OR study OR studies OR work)):ti,ab	46,386
28.	((mixed OR multi) NEAR/1 (method* OR stud*)):ti,ab	45,669
29.	*21-28 (OR)*	*3,751,998*
**Limits: publication type**		
30.	([conference abstract]/lim OR [conference paper]/lim OR [conference review]/lim OR [data papers]/lim OR [note]/lim OR [short survey]/lim OR [preprint]/lim)	6,734,6724
**Combined sets**		
31.	*4 AND (13 OR 16)*	*16,397*

32.	*19 OR (20 AND 31)*	*6,985*
33.	*(29 AND 32) NOT 30*	*2,789*
**Final result**		
**34.**	**33 AND ([danish]/lim OR [english]/lim OR [norwegian]/lim OR [swedish]/lim)**	**2,705**

The final search result, usually found at the end of the documentation, forms the list of abstracts.

/de = Term from the EMTREE controlled vocabulary; **/exp** = Includes terms found below this term in the EMTREE hierarchy **/mj** = Major Topic; **:ab** = Abstract; **:au** = Author; **:ti** = Article Title; **:ti,ab** = Title or abstract; ***** = Truncation; **' '** = Citation Marks; searches for an exact phrase; **NEAR/n** = Requests terms that are within 'n' words of each other in either direction; **NEXT/n** = Requests terms that are within 'n' words of each other in the order specified

Medline via OvidSP 27 June 2022

Title: Postpartum depression – patients experiences of care (qualitative studies)

Search terms		Items found
**Population: Postpartum depression**		
1.	Depression, Postpartum/	6883
2.	((postpart* or "post part*" or postnatal* or "post natal*" or peripart* or "peri part*" or perinatal or "peri natal" or postpregnan* or "post pregnan*" or maternal* or mother*) adj5 (affective or depress* or distress* or "mood disorder*")).ab,ti,kf.	19739
3.	Postpartum Period/	29264
4.	depressive disorder/ or depressive disorder, major/ or depressive disorder, treatment-resistant/ or dysthymic disorder/ or depression/	239302
5.	3 and 4	986
6.	*1 or 2 or 5*	21026
**Intervention: Psychological therapies, counseling, support**		
7.	exp Psychotherapy/	212639
8.	exp Counseling/	47673
9.	Home Care Services/	35678
10.	peer group/	23199
11.	(abreaction or "acceptance and commitment" or ACT or "applied behav*" or CBT or consultation* or counsel* or "crisis intervention" or DBT or "emotion focused" or "free association" or hypnosis or hypnotherapy or intervent* or IPT or "life review" or "listening visit*" or logotherap* or mindfulness or "mom power" or "motivational interview*" or "peer group" or "peer support" or "problem solv*" or program* or psychoanaly* or "psycho-analy*" or psychodynamic* or "psycho dynamic*" or psychodrama or "psycho-drama*" or psychoeducat* or "psychologic desenzitization" or "psychological feedback" or psychosocial or psychotherap* or "psycho therap*" or "rational emotive" or "reminiscence" or "self control" or "self management" or "self system" or "socioenvironmental therapy" or support* or therap* or "transactional analysis" or train* or treat*).ab,ti,kf.	10915460
12.	(behav* adj3 (activat* or component? or contracting or defusion or guidance or management or modif* or rehab* or restructur* or technique* or train*)).ab,ti,kf.	45457
13.	((cognitiv* or dialect*) adj3 (behav* or component? or contracting or defusion or guidance or management or modif* or rehab* or restructur* or technique* or train*)).ab,ti,kf.	66965
14.	((home or house) adj3 (based or call* or care or service* or visit*)).ab,ti,kf.	62878
15.	exp social support/	77848
16.	Self Care/	35393
17.	Self-Management/	4616
18.	Self-Help Groups/	9481
19.	((self-care or selfcare or Self-help or selfhelp) adj3 (administered or guided or instruct* or strateg* or supervised or tool*)).ab,kf,ti.	1927
20.	((self-administered or selfadministered) adj3 (guided or instruct* or strateg* or supervised)).ab,kf,ti.	103

21.	Patient Education as Topic/	88079
22.	*7 or 8 or 9 or 10 or 11 or 12 or 13 or 14 or 15 or 16 or 17 or 18 or 19 or 20 or 21*	11103771
**Intervention: Psychological therapies, counseling, support delivered via internet**		
23.	Computer-Assisted Instruction/	12410
24.	Therapy, Computer-Assisted/	6960
25.	Internet-Based Intervention/	964
26.	Telemedicine/	34017
27.	Remote Consultation/	5555
28.	Mobile Applications/	10202
29.	exp Internet/	92956
30.	exp Cell Phone/	20555
31.	Videoconferencing/	2241
32.	Telecommunications/	5014
33.	exp Computers, Handheld/	11749
34.	Medical Informatics Applications/	2550
35.	(cybertherapy or "e-aid" or "e-counsel*" or "e-health program" or ehealth or "e-mental health" or "e-psychotherapy" or "e-psychology" or "e-therapy" or "guided self-help" or "online clinical work" or "self-help through the internet" or telecounseling or telepsychiatry or telepsychology or teletherapy or cCBT or c-CBT or "cyber-counseling" or "cyber-counsel*" or cybercounsel* or "digital health" or "e-consultation" or eCBT or e-CBT or econsultation or "e-counsel*" or ecounsel* or "e-health" or ehealth or emedicine or "e-medicine" or "emental health*" or "e-mental health*" or "e-portal" or eportal or epsych* or "e-psych*" or "e-therap*" or etherap* or "i-CBT" or ICBT or "m-health" or mhealth or "mobile health" or Deprexis or Interapy or WeChat*).ab,ti,kf.	30277
36.	23 or 24 or 25 or 26 or 27 or 28 or 29 or 30 or 31 or 32 or 33 or 34 or 35	188248
**Evaluation: Patients experiences of care**		
37.	attitude/ or exp attitude to health/ or exp "treatment adherence and compliance"/ or exp "patient acceptance of health care"/ or exp patient compliance/ or exp medication adherence/ or exp directly observed therapy/ or exp no-show patients/ or exp patient dropouts/ or exp patient participation/ or exp patient satisfaction/ or treatment refusal/	510607
38.	((client* or patient* or women* or mother* or matern* or mum* or outpatient*) adj15 (accept* or attitude* or belief* or believ* or disbelief* or disbeliev* or dissatisf* or encounter* or expectation* or experience* or improvement* or interpret* or involvement or meaning or percept* or perspective* or satisf* or stigma* or relation or "self-report" or "treatment barrier*" or trust* or understanding* or unsatisf* or value or view*)).ab,kf,ti.	1444811
39.	*37 OR 38*	*1814437*
40.	((postpart* or "post part*" or postnatal* or "post natal*" or peripart* or "peri part*" or perinatal or "peri natal" or postpregnan* or "post pregnan*") adj5 (affective or depress* or distress* or "mood disorder*") adj15 (accept* or attitude* or belief* or believ* or disbelief* or disbeliev* or dissatisf* or encounter* or expectation* or experience* or improvement* or interpret* or involvement or meaning or percept* or perspective* or satisf* or stigma* or relation or "self-report" or "treatment barrier*" or trust* or understanding* or unsatisf* or value or view*)).ab,ti.	2176
**Study types: qualitative studies**		
41.	empirical research/ or grounded theory/ or qualitative research/	79852
42.	focus groups/ or interviews as topic/ or narration/ or "surveys and questionnaires"/	625955
43.	Interview, Psychological/	15267
44.	nursing research/ or nursing methodology research/	31705
45.	personal narratives as topic/	353
46.	Anecdotes as Topic/	4746
47.	exp tape recording/	15831
48.	exp video recording/	44067
49.	patient reported outcome measures/	11811

50.	(qualitative* or interview* or "focus group*" or phenomeno* or ethnolog* or ethnograph* or ethnonurs* or ethnomethodolog* or "meta-ethnograph*" or hermeneutic* or "grounded theory" or observation or "lived experience*" or narrat* or "mixed method*" or "content analys*" or "purposive sample*" or "thematic analys*" or "theoretical sample*" or "triangulation design").ab,kf,ti.	1311968
51.	((field adj2 (research or study or studies or work)) or (grounded adj2 (theor* or study or studies or research or analys?s)) or ((lived or life) adj2 (experience* or story or stories)) or ((video or tape) adj2 record*) or (("semi-structured" or semistructured or unstructured or informal or "in-depth" or indepth or "face-to-face" or structured or guide) adj3 (interview* or discussion* or questionnaire*))).ab,kf,ti.	239950
52.	("action research" or "cluster sample*" or "constant comparative method*" or "key informant*" or "maximum variation sampling*" or ((mixed or multi) adj1 (method* or stud*))).ab,kf,ti.	56229
53.	*41 or 42 or 43 or 44 or 45 or 46 or 47 or 48 or 49 OR 50 OR 51 OR 52*	*1927627*
**Combined sets**		
54.	*(6 AND 39) OR 40*	*7404*
55.	*22 OR 36*	*11192941*
56.	*53 AND 54 AND 55*	*2136*
**Final result**		
**57.**	**56**<br>**Limit to (danish or english or norwegian or swedish)**	**2075**

The final search result, usually found at the end of the documentation, forms the list of abstracts.

.ab. = Abstract; **.ab,ti.** = Abstract or title; **.af.** = All fields; **Exp** = Term from the Medline controlled vocabulary, including terms found below this term in the MeSH hierarchy; **.sh.** = Term from the Medline controlled vocabulary; **.ti.** = Title; **/** = Term from the Medline controlled vocabulary, but does not include terms found below this term in the MeSH hierarchy; ***** = Focus (if found in front of a MeSH-term); *** or $** = Truncation (if found at the end of a free text term); **.mp** = Text, heading word, subject area node, title; **" "** = Citation Marks; searches for an exact phrase; *ADJn* = Positional operator that lets you retrieve records that contain your terms (in any order) within a specified number (n) of words of each other.

PsycINFO via EBSCO 27 June 2022

Title: Postpartum depression – patients experiences of care (qualitative studies)

Search terms		Items found
**Population: Postpartum depression**		
1.	DE "Postpartum Depression"	5,493
2.	TI (((postpart* or "post part*" or postnatal* or "post natal*" or peripart* or "peri part*" or perinatal or "peri natal" or postpregnan* or "post pregnan*" or maternal* or mother*) n5 (affective or depress* or distress* or "mood disorder*"))) OR AB (((postpart* or "post part*" or postnatal* or "post natal*" or peripart* or "peri part*" or perinatal or "peri natal" or postpregnan* or "post pregnan*" or maternal* or mother*) n5 (affective or depress* or distress* or "mood disorder*"))) OR SU (((postpart* or "post part*" or postnatal* or "post natal*" or peripart* or "peri part*" or perinatal or "peri natal" or postpregnan* or "post pregnan*" or maternal* or mother*) n5 (affective or depress* or distress* or "mood disorder*")))	18,762
3.	DE "Postnatal Period" OR DE "Perinatal Period"	9,178
4.	DE "Major Depression" OR DE "Reactive Depression" OR DE "Recurrent Depression" OR DE "Treatment Resistant Depression" OR DE "Depression (Emotion)" OR DE "Dysthymic Disorder"	168,346
5.	3 and 4	870
6.	*1 or 2 or 5*	*18,868*
**Intervention: Psychological therapies, counseling, support**		
7.	DE "Psychotherapy" OR DE "Adlerian Psychotherapy" OR DE "Adolescent Psychotherapy" OR DE "Affirmative Therapy" OR DE "Analytical Psychotherapy" OR DE "Autogenic Training"	408,220

	OR DE "Brief Psychotherapy" OR DE "Brief Relational Therapy" OR DE "Child Psychotherapy" OR DE "Client Centered Therapy" OR DE "Conversion Therapy" OR DE "Couples Therapy" OR DE "Eclectic Psychotherapy" OR DE "Emotion Focused Therapy" OR DE "Existential Therapy" OR DE "Experiential Psychotherapy" OR DE "Expressive Psychotherapy" OR DE "Eye Movement Desensitization Therapy" OR DE "Feminist Therapy" OR DE "Geriatric Psychotherapy" OR DE "Gestalt Therapy" OR DE "Group Psychotherapy" OR DE "Guided Imagery" OR DE "Humanistic Psychotherapy" OR DE "Hypnotherapy" OR DE "Individual Psychotherapy" OR DE "Insight Therapy" OR DE "Integrative Psychotherapy" OR DE "Interpersonal Psychotherapy" OR DE "Logotherapy" OR DE "Narrative Therapy" OR DE "Network Therapy" OR DE "Persuasion Therapy" OR DE "Primal Therapy" OR DE "Psychoanalysis" OR DE "Psychodrama" OR DE "Psychodynamic Psychotherapy" OR DE "Psychotherapeutic Counseling" OR DE "Psychotherapeutic Techniques" OR DE "Rational Emotive Behavior Therapy" OR DE "Reality Therapy" OR DE "Relationship Therapy" OR DE "Solution Focused Therapy" OR DE "Strategic Therapy" OR DE "Supportive Psychotherapy" OR DE "Transactional Analysis" OR DE "Counseling" OR DE "Community Counseling" OR DE "Cross Cultural Counseling" OR DE "Educational Counseling" OR DE "Genetic Counseling" OR DE "Gerontological Counseling" OR DE "Grief Counseling" OR DE "Group Counseling" OR DE "Marriage Counseling" OR DE "Microcounseling" OR DE "Multicultural Counseling" OR DE "Occupational Guidance" OR DE "Pastoral Counseling" OR DE "Peer Counseling" OR DE "Premarital Counseling" OR DE "Psychotherapeutic Counseling" OR DE "Rehabilitation Counseling" OR DE "School Counseling" OR DE "Peer Counseling" OR DE "Community Services" OR DE "Community Mental Health Services" OR DE "Community Counseling" OR DE "Home Care" OR DE "Home Visiting Programs" OR DE "Social Support" OR DE "Support Groups" OR DE "Social Networks" OR DE "Self-Care" OR DE "Self-Management" OR DE "Self-Instructional Training" OR DE "Self-Help Techniques" OR DE "Client Education" OR DE "Disease Management" OR DE "Health Education"	
8.	TI ((abreaction or "acceptance and commitment" or ACT or "applied behav*" or CBT or consultation* or counsel* or "crisis intervention" or DBT or "emotion focused" or "free association" or hypnosis or hypnotherapy or intervent* or IPT or "life review" or "listening visit*" or logotherap* or mindfulness or "mom power" or "motivational interview*" or "peer group" or "peer support" or "problem solv*" or program* or psychoanaly* or "psycho-analy*" or psychodynamic* or "psycho dynamic*" or psychodrama or "psycho-drama*" or psychoeducat* or "psychologic desenzitization" or "psychological feedback" or psychosocial or psychotherap* or "psycho therap*" or "rational emotive" or "reminiscence" or "self control" or "self management" or "self system" or "socioenvironmental therapy" or support* or therap* or "transactional analysis" or train* or treat*)) OR AB ((abreaction or "acceptance and commitment" or ACT or "applied behav*" or CBT or consultation* or counsel* or "crisis intervention" or DBT or "emotion focused" or "free association" or hypnosis or hypnotherapy or intervent* or IPT or "life review" or "listening visit*" or logotherap* or mindfulness or "mom power" or "motivational interview*" or "peer group" or "peer support" or "problem solv*" or program* or psychoanaly* or "psycho-analy*" or psychodynamic* or "psycho dynamic*" or psychodrama or "psycho-drama*" or psychoeducat* or "psychologic desenzitization" or "psychological feedback" or psychosocial or psychotherap* or "psycho therap*" or "rational emotive" or "reminiscence" or "self control" or "self management" or "self system" or "socioenvironmental therapy" or support* or therap* or "transactional analysis" or train* or treat*)) OR SU ((abreaction or "acceptance and commitment" or ACT or "applied behav*" or CBT or consultation* or counsel* or "crisis intervention" or DBT or "emotion focused" or "free association" or hypnosis or hypnotherapy or intervent* or IPT or "life review" or "listening visit*" or logotherap* or mindfulness or "mom power" or "motivational interview*" or "peer group" or "peer support" or "problem solv*" or program* or psychoanaly* or "psycho-analy*" or psychodynamic* or "psycho dynamic*" or psychodrama or "psycho-drama*" or psychoeducat* or "psychologic desenzitization" or "psychological feedback" or psychosocial or psychotherap* or "psycho therap*" or "rational emotive" or "reminiscence" or "self control" or "self management" or "self system" or "socioenvironmental therapy" or support* or therap* or "transactional analysis" or train* or treat*))	2,594,379
9.	TI ((behav* n3 (activat* or component? or contracting or defusion or guidance or management or modif* or rehab* or restructur* or technique* or train*))) OR AB ((behav* n3 (activat* or component? or contracting or defusion or guidance or management or	64,127

	modif* or rehab* or restructur* or technique* or train*))) OR SU ((behav* n3 (activat* or component? or contracting or defusion or guidance or management or modif* or rehab* or restructur* or technique* or train*)))	
10.	TI (((cognitiv* or dialect*) n3 (behav* or component? or contracting or defusion or guidance or management or modif* or rehab* or restructur* or technique* or train*))) OR AB (((cognitiv* or dialect*) n3 (behav* or component? or contracting or defusion or guidance or management or modif* or rehab* or restructur* or technique* or train*))) OR SU (((cognitiv* or dialect*) n3 (behav* or component? or contracting or defusion or guidance or management or modif* or rehab* or restructur* or technique* or train*)))	99,770
11.	TI (((home or house) n3 (based or call* or care or service* or visit*))) OR AB (((home or house) n3 (based or call* or care or service* or visit*))) OR SU (((home or house) n3 (based or call* or care or service* or visit*)))	33,619
12.	TI (((self-care or selfcare or Self-help or selfhelp) n3 (administered or guided or instruct* or strateg* or supervised or tool*))) OR AB (((self-care or selfcare or Self-help or selfhelp) n3 (administered or guided or instruct* or strateg* or supervised or tool*))) OR SU (((self-care or selfcare or Self-help or selfhelp) n3 (administered or guided or instruct* or strateg* or supervised or tool*)))	1,707
13.	TI (((self-administered or selfadministered) n3 (guided or instruct* or strateg* or supervised))) OR AB (((self-administered or selfadministered) n3 (guided or instruct* or strateg* or supervised))) OR SU (((self-administered or selfadministered) n3 (guided or instruct* or strateg* or supervised)))	59
14.	*7 OR 8 OR 9 OR 10 OR 11 OR 12 OR 13*	*2,654,642*
**Intervention: Psychological therapies, counseling, support delivered via internet**		
15.	(DE "Computer Assisted Therapy" OR DE "Telemedicine" OR DE "Online Therapy" OR DE "Teleconsultation" OR DE "Telepsychiatry" OR DE "Telepsychology" OR DE "Computer Assisted Instruction" OR DE "Digital Interventions" OR DE "Electronic Health Services" OR DE "Mobile Health")	32,415
16.	TI ((cCBT or c-CBT or cyber-counseling or cyber-counselling or cybercounseling or cybercounselling or "digital health" or e-consultation or eCBT or e-CBT or econsultation or e-counseling or e-counselling or ecounseling or ecounselling or e-health or ehealth or emedicine or e-medicine or "emental health*" or "e-mental health*" or e-portal or eportal or epsych* or e-psych* or e-therapy or etherapy or i-CBT or ICBT or m-health or mhealth or "mobile health" or Deprexis or Interapy or WeChat*)) OR AB ((cCBT or c-CBT or cyber-counseling or cyber-counselling or cybercounseling or cybercounselling or "digital health" or e-consultation or eCBT or e-CBT or econsultation or e-counseling or e-counselling or ecounseling or ecounselling or e-health or ehealth or emedicine or e-medicine or "emental health*" or "e-mental health*" or e-portal or eportal or epsych* or e-psych* or e-therapy or etherapy or i-CBT or ICBT or m-health or mhealth or "mobile health" or Deprexis or Interapy or WeChat*)) OR KW ((cCBT or c-CBT or cyber-counseling or cyber-counselling or cybercounseling or cybercounselling or "digital health" or e-consultation or eCBT or e-CBT or econsultation or e-counseling or e-counselling or ecounseling or ecounselling or e-health or ehealth or emedicine or e-medicine or "emental health*" or "e-mental health*" or e-portal or eportal or epsych* or e-psych* or e-therapy or etherapy or i-CBT or ICBT or m-health or mhealth or "mobile health" or Deprexis or Interapy or WeChat*))	6,820
17.	*15 OR 16*	*35,095*
**Evaluation: Patients experiences of care**		
18.	((((DE "Client Attitudes" OR DE "Client Satisfaction" OR DE "Treatment Barriers") OR (DE "Consumer Satisfaction")) OR (DE "Preferences")) OR (DE "Dissatisfaction")) OR (DE "Perception")	92,764
19.	TI (((client* or patient* or women* or mother* or matern* or mum* or outpatient*) N15 (accept* or attitude* or belief* or believ* or disbelief* or disbeliev* or dissatisf* or encounter* or expectation* or experience* or improvement* or interpret* or involvement or meaning or percept* or perspective* or satisf* or stigma* or relation or "self-report" or " or treatment barrier* or trust* or understanding* or unsatisf* or value or view*))) OR AB (((client* or patient* or women* or mother* or matern* or mum* or outpatient*) N15 (accept* or attitude* or belief* or believ* or disbelief* or disbeliev* or dissatisf* or encounter* or expectation* or experience* or improvement* or interpret* or involvement or meaning or percept* or perspective* or satisf* or stigma* or relation or "self-report" or "	433,815

	or treatment barrier* or trust* or understanding* or unsatisf* or value or view*))) OR SU (((client* or patient* or women* or mother* or matern* or mum* or outpatient*) N15 (accept* or attitude* or belief* or believ* or disbelief* or disbeliev* or dissatisf* or encounter* or expectation* or experience* or improvement* or interpret* or involvement or meaning or percept* or perspective* or satisf* or stigma* or relation or "self-report" or " or treatment barrier* or trust* or understanding* or unsatisf* or value or view*))	
20.	*18 OR 19*	*494,303*
21.	TI ((postpart* or "post part*" or postnatal* or "post natal*" or peripart* or "peri part*" or perinatal or "peri natal" or postpregnan* or "post pregnan*") N5 (affective or depress* or distress* or "mood disorder*")) N15 (accept* or attitude* or belief* or believ* or disbelief* or disbeliev* or dissatisf* or encounter* or expectation* or experience* or improvement* or interpret* or involvement or meaning or percept* or perspective* or satisf* or stigma* or relation or "self-report" or "treatment barrier*" or trust* or understanding* or unsatisf* or value or view*) OR AB ((postpart* or "post part*" or postnatal* or "post natal*" or peripart* or "peri part*" or perinatal or "peri natal" or postpregnan* or "post pregnan*") N5 (affective or depress* or distress* or "mood disorder*")) N15 (accept* or attitude* or belief* or believ* or disbelief* or disbeliev* or dissatisf* or encounter* or expectation* or experience* or improvement* or interpret* or involvement or meaning or percept* or perspective* or satisf* or stigma* or relation or "self-report" or "treatment barrier*" or trust* or understanding* or unsatisf* or value or view*) OR SU ((postpart* or "post part*" or postnatal* or "post natal*" or peripart* or "peri part*" or perinatal or "peri natal" or postpregnan* or "post pregnan*") N5 (affective or depress* or distress* or "mood disorder*")) N15 (accept* or attitude* or belief* or believ* or disbelief* or disbeliev* or dissatisf* or encounter* or expectation* or experience* or improvement* or interpret* or involvement or meaning or percept* or perspective* or satisf* or stigma* or relation or "self-report" or "treatment barrier*" or trust* or understanding* or unsatisf* or value or view*)	1,997
**Study types: qualitative study design**		
22.	DE "Focus Group" OR DE "Grounded Theory" OR DE "Interpretative Phenomenological Analysis" OR DE "Narrative Analysis" OR DE "Semi-Structured Interview" OR DE "Thematic Analysis" OR DE "Interviews" OR DE "Mixed Methods Research" OR DE "Phenomenology" OR DE "Qualitative Measures" OR DE "Focus Group Interview" OR DE "Interpretative Phenomenological Analysis" OR DE "Attitude Measures" OR DE "Preference Measures" OR DE "Questionnaires" OR DE "Surveys" OR DE "Tape Recorders" OR DE "Videotape Recorders" OR DE "Content Analysis" OR DE "Digital Content Analysis" OR DE "Sentiment Analysis" OR DE "Social Network Analysis" OR DE "Cluster Analysis" OR DE "Ethnography" OR DE "Ethnology"	107,634
23.	TI (("action research" or "cluster sample*" or "constant comparative method*" or "key informant*" or "maximum variation sampling*" or qualitative* or interview* or "focus group*" or phenomeno* or ethnolog* or ethnograph* or ethnonurs* or ethnomethodolog* or "meta-ethnograph*" or hermeneutic* or "grounded theory" or observation or "lived experience*" or narrat* or "content analys*" or "purposive sample*" or "thematic analys*" or "theoretical sample*" or "triangulation design")) OR AB (("action research" or "cluster sample*" or "constant comparative method*" or "key informant*" or "maximum variation sampling*" or qualitative* or interview* or "focus group*" or phenomeno* or ethnolog* or ethnograph* or ethnonurs* or ethnomethodolog* or "meta-ethnograph*" or hermeneutic* or "grounded theory" or observation or "lived experience*" or narrat* or "content analys*" or or "purposive sample*" or "thematic analys*" or "theoretical sample*" or "triangulation design")) OR SU (("action research" or "cluster sample*" or "constant comparative method*" or "key informant*" or "maximum variation sampling*" or qualitative* or interview* or "focus group*" or phenomeno* or ethnolog* or ethnograph* or ethnonurs* or ethnomethodolog* or "meta-ethnograph*" or hermeneutic* or "grounded theory" or observation or "lived experience*" or narrat* or "content analys*" or "purposive sample*" or "thematic analys*" or "theoretical sample*" or "triangulation design"))	802,423
24.	TI ((((field n2 (research or study or studies or work))) OR ((grounded n2 (theor* or study or studies or research or analys*))) OR (((lived or life) n2 (experience* or story or stories))) OR (((video or tape) n2 record*)) OR ((("semi-structured" or semistructured or unstructured or informal or "in-depth" or indepth or "face-to-face" or structured or guide)	254,174

	n3 (interview* or discussion* or open ended or questionnaire*)))) OR AB ((((field n2 (research or study or studies or work))) OR ((grounded n2 (theor* or study or studies or research or analys*))) OR (((lived or life) n2 (experience* or story or stories))) OR (((video or tape) n2 record*)) OR ((("semi-structured" or semistructured or unstructured or informal or "in-depth" or indepth or "face-to-face" or structured or guide) n3 (interview* or discussion* or open ended or questionnaire*)))) OR SU ((((field n2 (research or study or studies or work))) OR ((grounded n2 (theor* or study or studies or research or analys*))) OR (((lived or life) n2 (experience* or story or stories))) OR (((video or tape) n2 record*)) OR ((("semi-structured" or semistructured or unstructured or informal or "in-depth" or indepth or "face-to-face" or structured or guide) n3 (interview* or discussion* or open ended or questionnaire*))))	
25.	TI ((mixed or multi) N1 (method* or stud*)) OR AB ((mixed or multi) N1 (method* or stud*)) OR SU ((mixed or multi) N1 (method* or stud*))	42,147
26.	22 OR 23 OR 24 OR 25	938,166
**Combined sets:**		
27.	*((6 AND 20) OR 21)*	*9,904*
28.	*(14 OR 17)*	*2,662,966*
29.	*26 AND 27 AND 28*	*1,861*
**Final result**		
**30.**	**29**<br>**Limiters - Language: Danish, English, Norwegian, Swedish**	**1,767**

The final search result, usually found at the end of the documentation, forms the list of abstracts.

AB = Abstract; **AU** = Author; **DE** = Term from the thesaurus; **MH**= Exact Subject Heading from CINAHL Subject Headings; **MM** = Major Concept; **TI** = Title; **TX** = All Text. Performs a keyword search of all the database's searchable fields; **ZC** = Methodology Index; ***** = Truncation; **" "** = Citation Marks; searches for an exact phrase; **N** = Near Operator (N) finds the words if they are a maximum of x words apart from one another, regardless of the order in which they appear.; **W** = Within Operator (W) finds the words if they are within x words of one another, in the order in which you entered them.

Hälsoekonomi/Health economics

CINAHL via EBSCO 8 August 2022

Title: Postpartum depression – economic evaluations

Search terms		Items found
**Population: Postpartum depression**		
1.	(MH "Depression, Postpartum")	6,678
2.	TI (((postpart* or "post part*" or postnatal* or "post natal*" or peripart* or "peri part*" or perinatal or "peri natal" or postpregnan* or "post pregnan*" or maternal* or mother*) n5 (affective or depress* or distress* or "mood disorder*"))) OR AB (((postpart* or "post part*" or postnatal* or "post natal*" or peripart* or "peri part*" or perinatal or "peri natal" or postpregnan* or "post pregnan*" or maternal* or mother*) n5 (affective or depress* or distress* or "mood disorder*"))) OR SU (((postpart* or "post part*" or postnatal* or "post natal*" or peripart* or "peri part*" or perinatal or "peri natal" or postpregnan* or "post pregnan*" or maternal* or mother*) n5 (affective or depress* or distress* or "mood disorder*")))	14,706
3.	(MH "Postnatal Period")	11,225
4.	(MH "Depression+")	128,950
5.	S3 AND S4	1,356
6.	*S1 OR S2 OR S5*	*14,883*
**Intervention: Psychological therapies, counseling, support**		
7.	(MH "Psychotherapy+")	218,973
8.	(MH "Counseling+")	42,076
9.	(MH "Home Health Care")	25,119
10.	(MH "Home Visits") OR (MH "Psychiatric Home Care")	6,907
11.	(MH "Peer Group")	15,379
12.	TI ((abreaction or "acceptance and commitment" or ACT or "applied behav*" or CBT or consultation* or counsel* or "crisis intervention" or DBT or "emotion focused" or "free association" or hypnosis or hypnotherapy or intervent* or IPT or "life review" or "listening visit*" or logotherap* or mindfulness or "mom power" or "motivational interview*" or "peer group" or "peer support" or "problem solv*" or program* or psychoanaly* or "psycho-analy*" or psychodynamic* or "psycho dynamic*" or psychodrama or "psycho-drama*" or psychoeducat* or "psychologic desenzitization" or "psychological feedback" or psychosocial or psychotherap* or "psycho therap*" or "rational emotive" or "reminiscence" or "self control" or "self management" or "self system" or "socioenvironmental therapy" or support* or therap* or "transactional analysis" or train* or treat*)) OR AB ((abreaction or "acceptance and commitment" or ACT or "applied behav*" or CBT or consultation* or counsel* or "crisis intervention" or DBT or "emotion focused" or "free association" or hypnosis or hypnotherapy or intervent* or IPT or "life review" or "listening visit*" or logotherap* or mindfulness or "mom power" or "motivational interview*" or "peer group" or "peer support" or "problem solv*" or program* or psychoanaly* or "psycho-analy*" or psychodynamic* or "psycho dynamic*" or psychodrama or "psycho-drama*" or psychoeducat* or "psychologic desenzitization" or "psychological feedback" or psychosocial or psychotherap* or "psycho therap*" or "rational emotive" or "reminiscence" or "self control" or "self management" or "self system" or "socioenvironmental therapy" or support* or therap* or "transactional analysis" or train* or treat*)) OR SU ((abreaction or "acceptance and commitment" or ACT or "applied behav*" or CBT or consultation* or counsel* or "crisis intervention" or DBT or "emotion focused" or "free association" or hypnosis or hypnotherapy or intervent* or IPT or "life review" or "listening visit*" or logotherap* or mindfulness or "mom power" or "motivational interview*" or "peer group" or "peer support" or "problem solv*" or program* or psychoanaly* or "psycho-analy*" or psychodynamic* or "psycho dynamic*" or psychodrama or "psycho-drama*" or psychoeducat* or "psychologic desenzitization" or "psychological feedback" or psychosocial or psychotherap* or "psycho therap*" or "rational emotive" or "reminiscence" or "self	3,898,664

	control" or "self management" or "self system" or "socioenvironmental therapy" or support* or therap* or "transactional analysis" or train* or treat*))	
13.	TI ((behav* n3 (activat* or component? or contracting or defusion or guidance or management or modif* or rehab* or restructur* or technique* or train*))) OR AB ((behav* n3 (activat* or component? or contracting or defusion or guidance or management or modif* or rehab* or restructur* or technique* or train*))) OR SU ((behav* n3 (activat* or component? or contracting or defusion or guidance or management or modif* or rehab* or restructur* or technique* or train*)))	21,999
14.	TI (((cognitiv* or dialect*) n3 (behav* or component? or contracting or defusion or guidance or management or modif* or rehab* or restructur* or technique* or train*))) OR AB (((cognitiv* or dialect*) n3 (behav* or component? or contracting or defusion or guidance or management or modif* or rehab* or restructur* or technique* or train*))) OR SU (((cognitiv* or dialect*) n3 (behav* or component? or contracting or defusion or guidance or management or modif* or rehab* or restructur* or technique* or train*)))	33,625
15.	TI (((home or house) n3 (based or call* or care or service* or visit*))) OR AB (((home or house) n3 (based or call* or care or service* or visit*))) OR SU (((home or house) n3 (based or call* or care or service* or visit*)))	76,100
16.	(MH "Support, Psychosocial+")	97,274
17.	(MH "Self Care+")	58,086
18.	(MH "Self-Management")	2,207
19.	(MH "Support Groups+")	12,055
20.	TI (((self-care or selfcare or Self-help or selfhelp) n3 (administered or guided or instruct* or strateg* or supervised or tool*))) OR AB (((self-care or selfcare or Self-help or selfhelp) n3 (administered or guided or instruct* or strateg* or supervised or tool*))) OR SU (((self-care or selfcare or Self-help or selfhelp) n3 (administered or guided or instruct* or strateg* or supervised or tool*)))	1,680
21.	TI (((self-administered or selfadministered) n3 (guided or instruct* or strateg* or supervised))) OR AB (((self-administered or selfadministered) n3 (guided or instruct* or strateg* or supervised))) OR SU (((self-administered or selfadministered) n3 (guided or instruct* or strateg* or supervised)))	51
22.	(MH "Patient Education")	69,604
23.	*S7 OR S8 OR S9 OR S10 OR S11 OR S12 OR S13 OR S14 OR S15 OR S16 OR S17 OR S18 OR S19 OR S20 OR S21 OR S22*	*3,972,769*
**Intervention: Psychological therapies, counseling, support delivered via internet**		
24.	(MH "Computer Assisted Instruction")	8,241
25.	(MH "Therapy, Computer Assisted")	5,496
26.	(MH "Internet-Based Intervention")	484
27.	(MH "Telemedicine")	15,318
28.	(MH "Remote Consultation")	2,919
29.	(MH "Mobile Applications")	11,074
30.	(MH "Internet+")	165,053
31.	(MH "Cellular Phone+")	9,505
32.	(MH "Videoconferencing")	2,688
33.	(MH "Telecommunications")	2,621
34.	(MH "Computers, Hand-Held+")	8,324
35.	(MH "Medical Informatics")	5,519
36.	TI ((cybertherapy or "e-aid" or "e-counsel*" or "e-health program" or ehealth or "e-mental health" or "e-psychotherapy" or "e-psychology" or "e-therapy" or "guided self-help" or "online clinical work" or "self-help through the internet" or telecounseling or telepsychiatry or telepsychology or teletherapy or cCBT or c-CBT or "cyber-counseling" or "cyber-counsel*" or cybercounsel* or "digital health" or "e-consultation" or eCBT or e-CBT or econsultation or "e-counsel*" or ecounsel* or "e-health" or ehealth or emedicine or "e-medicine" or "emental health*" or "e-mental health*" or "e-portal" or eportal or epsych* or "e-psych*" or "e-therap*" or etherap* or "i-CBT" or ICBT or "m-health" or mhealth or "mobile health" or Deprexis or Interapy or WeChat*)) OR AB ((cybertherapy or "e-aid" or "e-counsel*" or "e-health program" or ehealth or "e-mental health" or "e-psychotherapy"	16,474

	or "e-psychology" or "e-therapy" or "guided self-help" or "online clinical work" or "self-help through the internet" or telecounseling or telepsychiatry or telepsychology or teletherapy or cCBT or c-CBT or "cyber-counseling" or "cyber-counsel*" or cybercounsel* or "digital health" or "e-consultation" or eCBT or e-CBT or econsultation or "e-counsel*" or ecounsel* or "e-health" or ehealth or emedicine or "e-medicine" or "emental health*" or "e-mental health*" or "e-portal" or eportal or epsych* or "e-psych*" or "e-therap*" or etherap* or "i-CBT" or ICBT or "m-health" or mhealth or "mobile health" or Deprexis or Interapy or WeChat*)) OR SU ((cybertherapy or "e-aid" or "e-counsel*" or "e-health program" or ehealth or "e-mental health" or "e-psychotherapy" or "e-psychology" or "e-therapy" or "guided self-help" or "online clinical work" or "self-help through the internet" or telecounseling or telepsychiatry or telepsychology or teletherapy or cCBT or c-CBT or "cyber-counseling" or "cyber-counsel*" or cybercounsel* or "digital health" or "e-consultation" or eCBT or e-CBT or econsultation or "e-counsel*" or ecounsel* or "e-health" or ehealth or emedicine or "e-medicine" or "emental health*" or "e-mental health*" or "e-portal" or eportal or epsych* or "e-psych*" or "e-therap*" or etherap* or "i-CBT" or ICBT or "m-health" or mhealth or "mobile health" or Deprexis or Interapy or WeChat*))	
37.	*S24 OR S25 OR S26 OR S27 OR S28 OR S29 OR S30 OR S31 OR S32 OR S33 OR S34 OR S35 OR S36*	*227,027*
**Combined sets:**		
38.	*23 OR 37*	*4,090,738*
39.	*6 AND 38*	*11,566*
**Health economic aspects /Economic aspects**		
40.	(MH "Economics" OR MH ‘Cost Benefit Analysis’ OR MH "Costs and Cost Analysis+" OR MH "Cost Control+" OR MH "Health Care Costs+" OR MH "Economic Aspects of Illness" OR MH "Economic Value of Life" OR MH "Economics, Dental" OR MH "Economics, Pharmaceutical" OR MH "Fees and Charges+" OR MH "Resource Allocation+" OR MH "Health Facility Costs" OR MH "Nursing Costs" MH "Contract Services" OR MH "Health Resource Allocation" OR MH "Health Resource Utilization" OR MH "Quality-Adjusted Life Years") OR (TI (economic* OR cost* OR price* OR pharmacoeconomic* OR (pharm* N2 economic*) OR (resource N2 allocat*) OR (willingness W2 pay) OR pricing OR fee OR fees) OR AB((economic* N2 (evaluat* OR analy* OR study OR studies OR effectiv* OR utilit* OR benefit* OR consequenc* OR compare* OR compari* OR saving* OR efficienc*)) OR cost OR costs OR costly OR costing OR price* OR pricing OR pharmacoeconomic* OR (pharm* N2 economic*) OR (resource N2 allocat*) OR (willingness W2 pay) OR fee OR fees) OR KW((economic OR economics OR (economic* W2 (evaluat* OR analy* OR study OR studies OR effectiv* OR utilit* OR benefit* OR consequenc* OR compare* OR compari* OR saving* OR efficienc*)) OR cost OR costs OR costly OR costing OR price* OR pricing OR pharmacoeconomic* OR (pharm* N2 economic*) OR (resource N2 allocat*) OR (willingness W2 pay) OR fee OR fees)) OR (TI(icer OR "quality adjusted life" OR qaly OR hui* OR "value of life" OR hrqol OR eq5d OR sf36 OR sf6d OR "short form*" OR markov OR ((utilit* OR preferenc* OR instrument*) N5 (hrql OR "quality of life" OR score* OR weight*)) OR AB(icer OR "quality adjusted life" OR qaly OR hui* OR "value of life" OR hrqol OR hrql OR eq5d OR sf36 OR sf6d OR markov) OR KW(icer OR "quality adjusted life" OR qaly OR hui* OR "value of life" OR hrqol OR hrql OR eq5d OR sf36 OR sf6d OR "short form*" OR markov OR ((utilit* OR preferenc* OR instrument*) N5 (hrql OR "quality of life" OR score* OR weight*))))	368,540
**Final result**		
**41.**	**39 AND 40**<br>**Limiters - Language: Danish, English, Norwegian, Swedish**	**465**

The final search result, usually found at the end of the documentation, forms the list of abstracts.

AB = Abstract; **AU** = Author; **DE** = Term from the thesaurus; **MH**= Exact Subject Heading from CINAHL Subject Headings; **MM** = Major Concept; **TI** = Title; **TX** = All Text. Performs a keyword search of all the database's searchable fields; **ZC** = Methodology Index; ***** = Truncation; **" "** = Citation Marks; searches for an exact phrase; **N** = Near Operator (N) finds the words if they are a maximum of x words apart from one another, regardless of the order in which they appear.; **W** = Within Operator (W) finds the words if they are within x words of one another, in the order in which you entered them.

CRD Database (DARE, NHS EED) via CRD 21 February 2022

Title: Postpartum depression- economic evaluations

Search terms		Items found
**Population: Postpartum depression**		
1.	MeSH DESCRIPTOR depression, postpartum EXPLODE ALL TREES	67
2.	MeSH DESCRIPTOR postpartum period	70
3.	MeSH DESCRIPTOR depression EXPLODE ALL TREES	639
4.	#2 AND #3	1
5.	((postpart* or "post part*" or postnatal* or "post natal*" or peripart* or "peri part*" or perinatal or "peri natal" or postpregnan* or "post pregnan*" or maternal* or mother*) NEAR5 (affective or depress* or distress* or "mood disorder*"))	145
**Final result**		
**6.**	**1 OR 4 OR 5**	**157**

The final search result, usually found at the end of the documentation, forms the list of abstracts.

TITLE-ABS-KEY = Title or abstract or keywords; **ALL =** All fields; **PRE/n =** "precedes by". The first term in the search must precede the second by a specified number of terms (n).; **W/n =** "Within". The terms in the search must be within a specified number of terms (n) in any order.; ***** = Truncation; **" "** = Citation Marks; searches for an exact phrase; **LIMIT-TO (SRCTYPE, "j"** = Limit to source type journal; **LIMIT-TO (DOCTYPE, "ar"** = Limit to document type article; **LIMIT-TO (DOCTYPE, "re"** = Limit to document type review

Embase via Elsevier 9 August 2022

Title: Postpartum depression – economic evaluations

Search terms		Items found
**Population: Postpartum depression**		
1.	'perinatal depression'/de OR 'postnatal depression'/exp	14,955
2.	'puerperium'/de AND 'depression'/de	1,642
3.	((postpart* OR 'post part*' OR postnatal* OR 'post natal*' OR peripart* OR 'peri part*' OR perinatal OR 'peri natal' OR postpregnan* OR 'post pregnan*' OR maternal* OR mother*) NEAR/3 (affective OR depress* OR distress* OR 'mood disorder*')):ti,ab	21,822
4.	*1 OR 2 OR 3*	*25,055*
**Intervention: Psychological therapies, counseling, support**		
5.	'psychotherapy'/exp OR 'counseling'/de OR 'directive counseling'/de OR 'e-counseling'/de OR 'family counseling'/de OR 'motivational interviewing'/de OR 'parent counseling'/de OR 'patient counseling'/de OR 'patient guidance'/de OR 'peer counseling'/de OR 'psychological counseling'/de or 'home care'/de OR 'home mental health care'/de OR 'home visit'/de OR 'social support'/exp OR 'psychosocial care'/de OR 'peer counseling'/de	606,474
6.	abreaction:ti,kw,ab OR 'acceptance and commitment':ti,kw,ab OR act:ti,kw,ab OR 'applied behav*':ti,kw,ab OR cbt:ti,kw,ab OR consultation*:ti,kw,ab OR counsel*:ti,kw,ab OR 'crisis intervention':ti,kw,ab OR dbt:ti,kw,ab OR 'emotion focused':ti,kw,ab OR 'free association':ti,kw,ab OR hypnosis:ti,kw,ab OR hypnotherapy:ti,kw,ab OR intervent*:ti,kw,ab OR ipt:ti,kw,ab OR 'life review':ti,kw,ab OR 'listening visit*':ti,kw,ab OR logotherap*:ti,kw,ab OR mindfulness:ti,kw,ab OR 'mom power':ti,kw,ab OR 'motivational interview*':ti,kw,ab OR 'peer group':ti,kw,ab OR 'peer support':ti,kw,ab OR 'problem solv*':ti,kw,ab OR program*:ti,kw,ab OR psychoanaly*:ti,kw,ab OR 'psycho-analy*':ti,kw,ab OR psychodynamic*:ti,kw,ab OR 'psycho dynamic*':ti,kw,ab OR psychodrama:ti,kw,ab OR 'psycho-drama*':ti,kw,ab OR psychoeducat*:ti,kw,ab OR 'psychologic desenzitization':ti,kw,ab OR 'psychological feedback':ti,kw,ab OR psychosocial:ti,kw,ab OR psychotherap*:ti,kw,ab OR 'psycho therap*':ti,kw,ab OR 'rational emotive':ti,kw,ab OR 'reminiscence':ti,kw,ab OR 'self control':ti,kw,ab OR 'self management':ti,kw,ab OR 'self system':ti,kw,ab OR 'socioenvironmental therapy':ti,kw,ab OR support*:ti,kw,ab OR therap*:ti,kw,ab OR 'transactional analysis':ti,kw,ab OR train*:ti,kw,ab OR treat*:ti,kw,ab	14,530,830

7.	(behav* NEAR/3 (activat* OR component? OR contracting OR defusion OR guidance OR management OR modif* OR rehab* OR restructur* OR technique* OR train*)):ti,ab,kw	54,859
8.	(cognitiv* OR dialect*) NEAR/3 (behav* OR component* OR contracting OR defusion OR guidance OR management OR modif* OR rehab* OR restructur* OR technique* OR train*)	112,024
9.	((home OR house) NEAR/3 (based OR call* OR care OR service* OR visit*)):ti,ab,kw	82,734
10.	(('self care' OR selfcare OR 'self help' OR selfhelp) NEAR/3 (administered OR guided OR instruct* OR strateg* OR supervised OR tool*)):ti,ab,kw	2,495
11.	(('self administered' OR selfadministered) NEAR/3 (guided OR instruct* OR strateg* OR supervised)):ti,ab,kw	151
12.	'self help'/de OR 'patient education'/exp OR 'self care'/de	193,169
13.	*5 OR 6 OR 7 OR 8 OR 9 OR 10 OR 11 OR 12*	*14,786,759*
**Intervention: Psychological therapies, counseling, support delivered via internet**		
14.	'internet'/de OR 'medical informatics'/de OR 'telemedicine'/de OR 'teleconsultation'/exp OR 'telepsychiatry'/de OR 'telepsychology'/de OR 'video consultation'/de OR 'mobile application'/exp OR 'mobile phone'/exp OR 'videoconferencing'/exp OR 'telecommunication'/de OR 'personal digital assistant'/exp OR 'computer'/de	341,425
15.	cybertherapy:ti,ab OR 'e aid':ti,ab OR 'e counseling':ti,ab OR ecounseling:ti,ab OR 'e-health program':ti,ab OR 'e-mental health':ti,ab OR 'e psychotherapy':ti,ab OR 'e psychology':ti,ab OR 'e therapy':ti,ab OR 'guided self-help':ti,ab OR 'online clinical work':ti,ab OR 'self-help through the internet':ti,ab OR telecounseling:ti,ab OR telepsychiatry:ti,ab OR telepsychology:ti,ab OR teletherapy:ti,ab OR ccbt:ti,ab OR 'c cbt':ti,ab OR 'cyber-counseling':ti,ab OR 'cyber-counsel*':ti,ab OR cybercounsel*:ti,ab OR 'digital health':ti,ab OR 'e consultation':ti,ab OR ecbt:ti,ab OR 'e cbt':ti,ab OR econsultation:ti,ab OR 'e counsel*':ti,ab OR ecounsel*:ti,ab OR 'e health':ti,ab OR ehealth:ti,ab OR emedicine:ti,ab OR 'e medicine':ti,ab OR 'emental health*':ti,ab OR 'e-mental health*':ti,ab OR 'e portal':ti,ab OR eportal:ti,ab OR epsych*:ti,ab OR 'e psych*':ti,ab OR 'e therap*':ti,ab OR etherap*:ti,ab OR 'i cbt':ti,ab OR icbt:ti,ab OR 'm health':ti,ab OR mhealth:ti,ab OR 'mobile health':ti,ab OR deprexis:ti,ab OR interapy:ti,ab OR wechat*:ti,ab	27,528
16.	*14 OR 15*	*355,574*
**Health economic aspects**		
17.	('health economics'/de OR 'economic evaluation'/exp OR 'health care cost'/exp OR 'pharmacoeconomics'/exp OR econom*:ab,ti OR cost:ab,ti OR costs:ab,ti OR costly:ab,ti OR costing:ab,ti OR price:ab,ti OR prices:ab,ti OR pricing:ab,ti OR pharmacoeconomic*:ab,ti OR (expenditure* NOT energy):ti,ab OR (value NEXT/2 money):ab,ti OR budget*:ab,ti) NOT ((metabolic NEXT/2 cost):ab,ti OR ((energy or oxygen) NEXT/2 cost):ab,ti OR ((energy or oxygen) NEAR/2 expenditure):ab,ti)	1,679,084
**Combined sets**		
18.	*13 OR 16*	*14,972,408*
19.	*4 AND 18*	*16,372*
20.	*17 AND 19*	*1,163*
**Final result**		
**21.**	**20 AND ([danish]/lim OR [english]/lim OR [norwegian]/lim OR [swedish]/lim)**	**1,138**

The final search result, usually found at the end of the documentation, forms the list of abstracts.

/de = Term from the EMTREE controlled vocabulary; **/exp** = Includes terms found below this term in the EMTREE hierarchy **/mj** = Major Topic; **:ab** = Abstract; **:au** = Author; **:ti** = Article Title; **:ti,ab** = Title or abstract; ***** = Truncation; **' '** = Citation Marks; searches for an exact phrase; **NEAR/n** = Requests terms that are within 'n' words of each other in either direction; **NEXT/n** = Requests terms that are within 'n' words of each other in the order specified

Medline via OvidSP 8 August 2022

Title: Postpartum depression – economic evaluations

Search terms		Items found
**Population: Postpartum depression**		
1.	Depression, Postpartum/	6983
2.	((postpart* or "post part*" or postnatal* or "post natal*" or peripart* or "peri part*" or perinatal or "peri natal" or postpregnan* or "post pregnan*" or maternal* or mother*) adj5 (affective or depress* or distress* or "mood disorder*")).ab,ti,kf.	19986
3.	Postpartum Period/	29473
4.	depressive disorder/ or depressive disorder, major/ or depressive disorder, treatment-resistant/ or dysthymic disorder/ or depression/	240474
5.	3 and 4	1000
6.	*1 or 2 or 5*	*21278*
**Intervention: Psychological therapies, counseling, support**		
7.	exp Psychotherapy/	213286
8.	exp Counseling/	47832
9.	Home Care Services/	35765
10.	peer group/	23337
11.	(abreaction or "acceptance and commitment" or ACT or "applied behav*" or CBT or consultation* or counsel* or "crisis intervention" or DBT or "emotion focused" or "free association" or hypnosis or hypnotherapy or intervent* or IPT or "life review" or "listening visit*" or logotherap* or mindfulness or "mom power" or "motivational interview*" or "peer group" or "peer support" or "problem solv*" or program* or psychoanaly* or "psycho-analy*" or psychodynamic* or "psycho dynamic*" or psychodrama or "psycho-drama*" or psychoeducat* or "psychologic desenzitization" or "psychological feedback" or psychosocial or psychotherap* or "psycho therap*" or "rational emotive" or "reminiscence" or "self control" or "self management" or "self system" or "socioenvironmental therapy" or support* or therap* or "transactional analysis" or train* or treat*).ab,ti,kf.	11003259
12.	(behav* adj3 (activat* or component? or contracting or defusion or guidance or management or modif* or rehab* or restructur* or technique* or train*)).ab,ti,kf.	45866
13.	((cognitiv* or dialect*) adj3 (behav* or component? or contracting or defusion or guidance or management or modif* or rehab* or restructur* or technique* or train*)).ab,ti,kf.	67663
14.	((home or house) adj3 (based or call* or care or service* or visit*)).ab,ti,kf.	63518
15.	exp social support/	78109
16.	Self Care/	35488
17.	Self-Management/	4733
18.	Self-Help Groups/	9490
19.	((self-care or selfcare or Self-help or selfhelp) adj3 (administered or guided or instruct* or strateg* or supervised or tool*)).ab,kf,ti.	1940
20.	((self-administered or selfadministered) adj3 (guided or instruct* or strateg* or supervised)).ab,kf,ti.	105
21.	Patient Education as Topic/	88112
22.	*7 or 8 or 9 or 10 or 11 or 12 or 13 or 14 or 15 or 16 or 17 or 18 or 19 or 20 or 21*	*11192114*
**Intervention: Psychological therapies, counseling, support delivered via internet**		
23.	Computer-Assisted Instruction/	12441
24.	Therapy, Computer-Assisted/	6961
25.	Internet-Based Intervention/	995
26.	Telemedicine/	34522
27.	Remote Consultation/	5578
28.	Mobile Applications/	10414
29.	exp Internet/	93559
30.	exp Cell Phone/	20799
31.	Videoconferencing/	2264

32.	Telecommunications/	5017
33.	exp Computers, Handheld/	11930
34.	Medical Informatics Applications/	2550
35.	(cybertherapy or "e-aid" or "e-counsel*" or "e-health program" or ehealth or "e-mental health" or "e-psychotherapy" or "e-psychology" or "e-therapy" or "guided self-help" or "online clinical work" or "self-help through the internet" or telecounseling or telepsychiatry or telepsychology or teletherapy or cCBT or c-CBT or "cyber-counseling" or "cyber-counsel*" or cybercounsel* or "digital health" or "e-consultation" or eCBT or e-CBT or econsultation or "e-counsel*" or ecounsel* or "e-health" or ehealth or emedicine or "e-medicine" or "emental health*" or "e-mental health*" or "e-portal" or eportal or epsych* or "e-psych*" or "e-therap*" or etherap* or "i-CBT" or ICBT or "m-health" or mhealth or "mobile health" or Deprexis or Interapy or WeChat*).ab,ti,kf.	31048
36.	*23 or 24 or 25 or 26 or 27 or 28 or 29 or 30 or 31 or 32 or 33 or 34 or 35*	*190252*
37.	*22 OR 36*	*11282038*
**Health economic aspects (filter: NHS EED[2]) /Economic aspects**		
38.	economics/ or exp "costs and cost analysis"/ or economics, dental/ or exp "economics, hospital"/ or economics, medical/ or economics, nursing/ or economics, pharmaceutical/	308216
39.	(economic$ or cost or costs or costly or costing or price or prices or pricing or pharmacoeconomic$).ab,ti.	963874
40.	(expenditure$ not energy).ab,ti.	34742
41.	((value adj1 money) or budget$).ab,ti.	33637
42.	((energy or oxygen) adj cost).ab,ti.	4586
43.	(metabolic adj cost).ab,ti.	1621
44.	((energy or oxygen) adj expenditure).ab,ti.	27930
45.	(letter or historical article).pt.	1550481
46.	42 or 43 or 44 or 45	1583354
47.	38 or 39 or 40 or 41	1125291
48.	*47 not 46*	*1093717*
**Combined sets**		
49.	6 and 37 and 48	836
**Final result**		
**50.**	limit 49 to (danish or english or norwegian or swedish)	**811**

The final search result, usually found at the end of the documentation, forms the list of abstracts.

.ab. = Abstract; .**ab,ti.** = Abstract or title; **.af.** = All fields; **Exp** = Term from the Medline controlled vocabulary, including terms found below this term in the MeSH hierarchy; **.sh.** = Term from the Medline controlled vocabulary; **.ti.** = Title; **/** = Term from the Medline controlled vocabulary, but does not include terms found below this term in the MeSH hierarchy; ***** = Focus (if found in front of a MeSH-term); *** or $** = Truncation (if found at the end of a free text term); **.mp** = Text, heading word, subject area node, title; **“ “** = Citation Marks; searches for an exact phrase; *ADJn* = Positional operator that lets you retrieve records that contain your terms (in any order) within a specified number (n) of words of each other.

[2] http://www.crd.york.ac.uk/crdweb/searchstrategies.asp

PsycINFO via EBSCO 15 February 2022

Title: Postpartum depression – economic evaluations

Search terms		Items found
**Population: Postpartum depression**		
1.	DE "Postpartum Depression" OR DE "Postpartum Psychosis"	5,532
2.	TI (((postpart* or "post part*" or postnatal* or "post natal*" or peripart* or "peri part*" or perinatal or "peri natal" or postpregnan* or "post pregnan*" or maternal* or mother*) n5 (affective or depress* or distress* or "mood disorder*"))) OR AB (((postpart* or "post part*" or postnatal* or "post natal*" or peripart* or "peri part*" or perinatal or "peri natal" or postpregnan* or "post pregnan*" or maternal* or mother*) n5 (affective or depress* or distress* or "mood disorder*"))) OR SU (((postpart* or "post part*" or postnatal* or "post natal*" or peripart* or "peri part*" or perinatal or "peri natal" or postpregnan* or "post pregnan*" or maternal* or mother*) n5 (affective or depress* or distress* or "mood disorder*")))	18,891
3.	DE "Postnatal Period" OR DE "Perinatal Period"	9,302
4.	DE "Major Depression" OR DE "Reactive Depression" OR DE "Recurrent Depression" OR DE "Treatment Resistant Depression" OR DE "Depression (Emotion)" OR DE "Dysthymic Disorder"	169,569
5.	3 and 4	889
6.	*1 or 2 or 5*	*19,000*
**Intervention: Psychological therapies, counseling, support**		
7.	DE "Psychotherapy" OR DE "Adlerian Psychotherapy" OR DE "Adolescent Psychotherapy" OR DE "Affirmative Therapy" OR DE "Analytical Psychotherapy" OR DE "Autogenic Training" OR DE "Brief Psychotherapy" OR DE "Brief Relational Therapy" OR DE "Child Psychotherapy" OR DE "Client Centered Therapy" OR DE "Conversion Therapy" OR DE "Couples Therapy" OR DE "Eclectic Psychotherapy" OR DE "Emotion Focused Therapy" OR DE "Existential Therapy" OR DE "Experiential Psychotherapy" OR DE "Expressive Psychotherapy" OR DE "Eye Movement Desensitization Therapy" OR DE "Feminist Therapy" OR DE "Geriatric Psychotherapy" OR DE "Gestalt Therapy" OR DE "Group Psychotherapy" OR DE "Guided Imagery" OR DE "Humanistic Psychotherapy" OR DE "Hypnotherapy" OR DE "Individual Psychotherapy" OR DE "Insight Therapy" OR DE "Integrative Psychotherapy" OR DE "Interpersonal Psychotherapy" OR DE "Logotherapy" OR DE "Narrative Therapy" OR DE "Network Therapy" OR DE "Persuasion Therapy" OR DE "Primal Therapy" OR DE "Psychoanalysis" OR DE "Psychodrama" OR DE "Psychodynamic Psychotherapy" OR DE "Psychotherapeutic Counseling" OR DE "Psychotherapeutic Techniques" OR DE "Rational Emotive Behavior Therapy" OR DE "Reality Therapy" OR DE "Relationship Therapy" OR DE "Solution Focused Therapy" OR DE "Strategic Therapy" OR DE "Supportive Psychotherapy" OR DE "Transactional Analysis"	200,831
8.	DE "Counseling" OR DE "Community Counseling" OR DE "Cross Cultural Counseling" OR DE "Educational Counseling" OR DE "Genetic Counseling" OR DE "Gerontological Counseling" OR DE "Grief Counseling" OR DE "Group Counseling" OR DE "Marriage Counseling" OR DE "Microcounseling" OR DE "Multicultural Counseling" OR DE "Occupational Guidance" OR DE "Pastoral Counseling" OR DE "Peer Counseling" OR DE "Premarital Counseling" OR DE "Psychotherapeutic Counseling" OR DE "Rehabilitation Counseling" OR DE "School Counseling"	67,654
9.	DE "Community Services" OR DE "Community Mental Health Services" OR DE "Community Counseling" OR DE "Home Care" OR DE "Home Visiting Programs"	40,570
10.	DE "Peer Counseling"	1,205
11.	TI ((abreaction or "acceptance and commitment" or ACT or "applied behav*" or CBT or consultation* or counsel* or "crisis intervention" or DBT or "emotion focused" or "free association" or hypnosis or hypnotherapy or intervent* or IPT or "life review" or "listening visit*" or logotherap* or mindfulness or "mom power" or "motivational interview*" or "peer group" or "peer support" or "problem solv*" or program* or psychoanaly* or "psycho-analy*" or psychodynamic* or "psycho dynamic*" or psychodrama or "psycho-drama*" or psychoeducat* or "psychologic desenzitization" or "psychological feedback" or psychosocial or psychotherap* or "psycho therap*" or "rational emotive" or "reminiscence" or "self control" or "self management" or "self system" or "socioenvironmental therapy" or	2,607,936

	support* or therap* or "transactional analysis" or train* or treat*)) OR AB ((abreaction or "acceptance and commitment" or ACT or "applied behav*" or CBT or consultation* or counsel* or "crisis intervention" or DBT or "emotion focused" or "free association" or hypnosis or hypnotherapy or intervent* or IPT or "life review" or "listening visit*" or logotherap* or mindfulness or "mom power" or "motivational interview*" or "peer group" or "peer support" or "problem solv*" or program* or psychoanaly* or "psycho-analy*" or psychodynamic* or "psycho dynamic*" or psychodrama or "psycho-drama*" or psychoeducat* or "psychologic desenzitization" or "psychological feedback" or psychosocial or psychotherap* or "psycho therap*" or "rational emotive" or "reminiscence" or "self control" or "self management" or "self system" or "socioenvironmental therapy" or support* or therap* or "transactional analysis" or train* or treat*)) OR SU ((abreaction or "acceptance and commitment" or ACT or "applied behav*" or CBT or consultation* or counsel* or "crisis intervention" or DBT or "emotion focused" or "free association" or hypnosis or hypnotherapy or intervent* or IPT or "life review" or "listening visit*" or logotherap* or mindfulness or "mom power" or "motivational interview*" or "peer group" or "peer support" or "problem solv*" or program* or psychoanaly* or "psycho-analy*" or psychodynamic* or "psycho dynamic*" or psychodrama or "psycho-drama*" or psychoeducat* or "psychologic desenzitization" or "psychological feedback" or psychosocial or psychotherap* or "psycho therap*" or "rational emotive" or "reminiscence" or "self control" or "self management" or "self system" or "socioenvironmental therapy" or support* or therap* or "transactional analysis" or train* or treat*))	
12.	TI ((behav* n3 (activat* or component? or contracting or defusion or guidance or management or modif* or rehab* or restructur* or technique* or train*))) OR AB ((behav* n3 (activat* or component? or contracting or defusion or guidance or management or modif* or rehab* or restructur* or technique* or train*))) OR SU ((behav* n3 (activat* or component? or contracting or defusion or guidance or management or modif* or rehab* or restructur* or technique* or train*)))	64,426
13.	TI (((cognitiv* or dialect*) n3 (behav* or component? or contracting or defusion or guidance or management or modif* or rehab* or restructur* or technique* or train*))) OR AB (((cognitiv* or dialect*) n3 (behav* or component? or contracting or defusion or guidance or management or modif* or rehab* or restructur* or technique* or train*))) OR SU (((cognitiv* or dialect*) n3 (behav* or component? or contracting or defusion or guidance or management or modif* or rehab* or restructur* or technique* or train*)))	100,439
14.	TI (((home or house) n3 (based or call* or care or service* or visit*))) OR AB (((home or house) n3 (based or call* or care or service* or visit*))) OR SU (((home or house) n3 (based or call* or care or service* or visit*)))	33,828
15.	DE "Social Support" OR DE "Support Groups" OR DE "Social Networks"	78,133
16.	DE "Self-Care"	3,359
17.	DE "Self-Management" OR DE "Self-Instructional Training"	8,048
18.	DE "Self-Help Techniques" OR DE "Self-Management"	12,196
19.	TI (((self-care or selfcare or Self-help or selfhelp) n3 (administered or guided or instruct* or strateg* or supervised or tool*))) OR AB (((self-care or selfcare or Self-help or selfhelp) n3 (administered or guided or instruct* or strateg* or supervised or tool*))) OR SU (((self-care or selfcare or Self-help or selfhelp) n3 (administered or guided or instruct* or strateg* or supervised or tool*)))	1,728
20.	TI (((self-administered or selfadministered) n3 (guided or instruct* or strateg* or supervised))) OR AB (((self-administered or selfadministered) n3 (guided or instruct* or strateg* or supervised))) OR SU (((self-administered or selfadministered) n3 (guided or instruct* or strateg* or supervised)))	59
21.	DE "Client Education" OR DE "Disease Management" OR DE "Health Education"	34,095
22.	*7 or 8 or 9 or 10 or 11 or 12 or 13 or 14 or 15 or 16 or 17 or 18 or 19 or 20 or 21*	*2,668,497*
**Intervention: Psychological therapies, counseling, support delivered via internet**		
23.	(DE "Computer Assisted Therapy" OR DE "Telemedicine" OR DE "Online Therapy" OR DE "Teleconsultation" OR DE "Telepsychiatry" OR DE "Telepsychology" OR DE "Computer Assisted Instruction" OR DE "Digital Interventions" OR DE "Electronic Health Services" OR DE "Mobile Health") OR (DE "Mobile Devices" OR DE "Mobile Phones" OR DE "Smartphones" OR DE "Tablet Computers" OR DE "Text Messaging" OR DE "Computer	32,802

	Mediated Communication" OR DE "Websites" OR DE "Internet" OR DE "Computer Applications" OR DE "Mobile Applications")	
24.	TI ((cCBT or c-CBT or cyber-counseling or cyber-counselling or cybercounseling or cybercounselling or "digital health" or e-consultation or eCBT or e-CBT or econsultation or e-counseling or e-counselling or ecounseling or ecounselling or e-health or ehealth or emedicine or e-medicine or "emental health*" or "e-mental health*" or e-portal or eportal or epsych* or e-psych* or e-therapy or etherapy or i-CBT or ICBT or m-health or mhealth or "mobile health" or Deprexis or Interapy or WeChat*)) OR AB ((cCBT or c-CBT or cyber-counseling or cyber-counselling or cybercounseling or cybercounselling or "digital health" or e-consultation or eCBT or e-CBT or econsultation or e-counseling or e-counselling or ecounseling or ecounselling or e-health or ehealth or emedicine or e-medicine or "emental health*" or "e-mental health*" or e-portal or eportal or epsych* or e-psych* or e-therapy or etherapy or i-CBT or ICBT or m-health or mhealth or "mobile health" or Deprexis or Interapy or WeChat*)) OR KW ((cCBT or c-CBT or cyber-counseling or cyber-counselling or cybercounseling or cybercounselling or "digital health" or e-consultation or eCBT or e-CBT or econsultation or e-counseling or e-counselling or ecounseling or ecounselling or e-health or ehealth or emedicine or e-medicine or "emental health*" or "e-mental health*" or e-portal or eportal or epsych* or e-psych* or e-therapy or etherapy or i-CBT or ICBT or m-health or mhealth or "mobile health" or Deprexis or Interapy or WeChat*))	6,945
25.	*23 OR 24*	*35,524*
**Combined sets:**		
26.	*22 OR 25*	*2,676,904*
27.	*6 AND 26*	*12,234*
**Health economic aspects /Economic aspects**		
28.	DE "Economics" OR DE "Health Care Economics" OR DE "Cost Containment" OR DE "Costs and Cost Analysis" OR DE "Health Care Costs" OR DE "Pharmacoeconomics" OR DE "Health Care Utilization" OR DE "Resource Allocation" OR (TI (economic* OR cost* OR price* OR pharmacoeconomic* OR (pharm* N2 economic*) OR (resource N2 allocat*) OR (willingness W2 pay) OR pricing OR fee OR fees) OR AB((economic* N2 (evaluat* OR analy* OR study OR studies OR effectiv* OR utilit* OR benefit* OR consequenc* OR compare* OR compari* OR saving* OR efficienc*)) OR cost OR costs OR costly OR costing OR price* OR pricing OR pharmacoeconomic* OR (pharm* N2 economic*) OR (resource N2 allocat*) OR (willingness W2 pay) OR fee OR fees) OR KW((economic OR economics OR (economic* W2 (evaluat* OR analy* OR study OR studies OR effectiv* OR utilit* OR benefit* OR consequenc* OR compare* OR compari* OR saving* OR efficienc*)) OR cost OR costs OR costly OR costing OR price* OR pricing OR pharmacoeconomic* OR (pharm* N2 economic*) OR (resource N2 allocat*) OR (willingness W2 pay) OR fee OR fees)) OR (TI(icer OR "quality adjusted life" OR qaly OR hui* OR "value of life" OR hrqol OR eq5d OR sf36 OR sf6d OR "short form*" OR markov OR ((utilit* OR preferenc* OR instrument*) N5 (hrql OR "quality of life" OR score* OR weight*)) OR AB(icer OR "quality adjusted life" OR qaly OR hui* OR "value of life" OR hrqol OR hrql OR eq5d OR sf36 OR sf6d OR markov) OR KW(icer OR "quality adjusted life" OR qaly OR hui* OR "value of life" OR hrqol OR hrql OR eq5d OR sf36 OR sf6d OR "short form*" OR markov OR ((utilit* OR preferenc* OR instrument*) N5 (hrql OR "quality of life" OR score* OR weight*))))	220,447
**Final result**		
**29.**	**27 AND 28**<br>**Limiters - Language: Danish, English, Norwegian, Swedish**	**444**

The final search result, usually found at the end of the documentation, forms the list of abstracts.

AB = Abstract; **AU** = Author; **DE** = Term from the thesaurus; **MH**= Exact Subject Heading from CINAHL Subject Headings; **MM** = Major Concept; **TI** = Title; **TX** = All Text. Performs a keyword search of all the database's searchable fields; **ZC** = Methodology Index; ***** = Truncation; **" "** = Citation Marks; searches for an exact phrase; **N** = Near Operator (N) finds the words if they are a maximum of x words apart from one another, regardless of the order in which they appear.; **W** = Within Operator (W) finds the words if they are within x words of one another, in the order in which you entered them.

Scopus via Elsevier 9 August 2022

Title: Postpartum depression- economic evaluations

Search terms		Items found
**Population: Postpartum depression**		
1.	TITLE-ABS-KEY ((postpart* OR "post part*" OR postnatal* OR "post natal*" OR peripart* OR "peri part*" OR perinatal OR "peri natal" OR postpregnan* OR "post pregnan*" OR maternal* OR mother*) W/3 (affective OR depress* OR distress* OR "mood disorder*"))	25,858
**Health economic aspects /economic aspects**		
2.	((TITLE-ABS-KEY (econom* OR cost OR costs OR costly OR costing OR price OR prices OR pricing OR pharmacoeconomic*) OR TITLE-ABS-KEY (expenditure* AND not AND energy) OR TITLE-ABS-KEY (value W/2 money) OR TITLE-ABS-KEY (budget*))) AND NOT ((TITLE-ABS-KEY (metabolic W/2 cost) OR TITLE-ABS-KEY ((energy OR oxygen) W/2 cost) OR TITLE-ABS-KEY ((energy OR oxygen) W/2 expenditure)))	5,934,091
**Combined sets**		
3.	1 AND 2	1,462
**Final result**		
**4.**	**3**<br>**(LIMIT-TO (LANGUAGE , "english") OR LIMIT-TO (LANGUAGE , "norwegian"))**	**1,466**

The final search result, usually found at the end of the documentation, forms the list of abstracts.

TITLE-ABS-KEY = Title or abstract or keywords; **ALL =** All fields; **PRE/n =** "precedes by". The first term in the search must precede the second by a specified number of terms (n).; **W/n =** "Within". The terms in the search must be within a specified number of terms (n) in any order.; ***** = Truncation; **" "** = Citation Marks; searches for an exact phrase; **LIMIT-TO (SRCTYPE, "j"** = Limit to source type journal; **LIMIT-TO (DOCTYPE, "ar"** = Limit to document type article; **LIMIT-TO (DOCTYPE, "re"** = Limit to document type review

International HTA Database via INAHTA 9 August 2022

Title: Postpartum depression- economic evaluations

Search terms		Items found
**Population: Postpartum depression**		
1.	"Depression, Postpartum"[mh]	9
2.	(((postpart* or "post part*" or postnatal* or "post natal*" or peripart* or "peri part*" or perinatal or "peri natal" or postpregnan* or "post pregnan*" or maternal* or mother*) AND (affective or depress* or distress* or "mood disorder*")))[abs]	23
3.	(((postpart* or "post part*" or postnatal* or "post natal*" or peripart* or "peri part*" or perinatal or "peri natal" or postpregnan* or "post pregnan*" or maternal* or mother*) AND (affective or depress* or distress* or "mood disorder*")))[keywords]	0
4.	(((postpart* or "post part*" or postnatal* or "post natal*" or peripart* or "peri part*" or perinatal or "peri natal" or postpregnan* or "post pregnan*" or maternal* or mother*) AND (affective or depress* or distress* or "mood disorder*")))[title]	11
5.	"Postpartum Period"[mh]	17
6.	"Depression"[mh]	131
7.	"Depressive Disorder"[mh]	134
8.	6 OR 7	211
9.	5 AND 8	2
**Final result**		
**10.**	**1 OR 2 OR 3 OR 4 OR 9**	**28**

The final search result, usually found at the end of the documentation, forms the list of abstracts.

TITLE-ABS-KEY = Title or abstract or keywords; **ALL =** All fields; **PRE/n =** "precedes by". The first term in the search must precede the second by a specified number of terms (n).; **W/n =** "Within". The terms in the search must be within a specified number of terms (n) in any order.; ***** = Truncation; **" "** = Citation Marks; searches for an exact phrase; **LIMIT-TO (SRCTYPE, "j"** = Limit to source type journal; **LIMIT-TO (DOCTYPE, "ar"** = Limit to document type article; **LIMIT-TO (DOCTYPE, "re"** = Limit to document type review
